# The tumor suppressor activity of DLC1 requires the interaction of its START domain with Phosphatidylserine, PLCD1, and Caveolin-1

**DOI:** 10.1186/s12943-021-01439-y

**Published:** 2021-11-02

**Authors:** Beatriz Sanchez-Solana, Dunrui Wang, Xiaolan Qian, Parthibane Velayoudame, Dhirendra K. Simanshu, Jairaj K. Acharya, Douglas R. Lowy

**Affiliations:** 1 Laboratory of Cellular Oncology, Center for Cancer Research, National Cancer Institute, National Institutes of Health, Bethesda, MD 20892 USA; 2 Cancer and Developmental Biology Laboratory, Center for Cancer Research, National Cancer Institute, NIH, Frederick, MD 21701 USA; 3 NCI RAS Initiative, Cancer Research Technology Program, Frederick National Laboratory for Cancer Research, Leidos Biomedical Research, Frederick, MD 21701 USA

**Keywords:** DLC1, Tumor suppressor, Rho-GAP, Phosphatidylserine, PLCD1, Caveolin-1, Protein-protein interactions, Lipid-binding domain

## Abstract

**Background:**

*DLC1*, a tumor suppressor gene that is downregulated in many cancer types by genetic and nongenetic mechanisms, encodes a protein whose RhoGAP and scaffolding activities contribute to its tumor suppressor functions. The role of the DLC1 START (StAR-related lipid transfer; DLC1-START) domain, other than its binding to Caveolin-1, is poorly understood. In other START domains, a key function is that they bind lipids, but the putative lipid ligand for DLC1-START is unknown.

**Methods:**

Lipid overlay assays and Phosphatidylserine (PS)-pull down assays confirmed the binding of DLC1-START to PS. Co-immunoprecipitation studies demonstrated the interaction between DLC1-START and Phospholipase C delta 1 (PLCD1) or Caveolin-1, and the contribution of PS to those interactions. Rho-GTP, cell proliferation, cell migration, and/or anchorage-independent growth assays were used to investigate the contribution of PS and PLCD1, or the implications of TCGA cancer-associated DLC1-START mutants, to DLC1 functions. Co-immunoprecipitations and PS-pull down assays were used to investigate the molecular mechanisms underlying the impaired functions of DLC1-START mutants. A structural model of DLC1-START was also built to better understand the structural implications of the cancer-associated mutations in DLC1-START.

**Results:**

We identified PS as the lipid ligand for DLC1-START and determined that DLC1-START also binds PLCD1 protein in addition to Caveolin-1. PS binding contributes to the interaction of DLC1 with Caveolin-1 and with PLCD1. The importance of these activities for tumorigenesis is supported by our analysis of 7 cancer-associated DLC1-START mutants, each of which has reduced tumor suppressor function but retains wildtype RhoGAP activity. Our structural model of DLC1-START indicates the mutants perturb different elements within the structure, which is correlated with our experimental findings that the mutants are heterogenous with regard to the deficiency of their binding properties. Some have reduced PS binding, others reduced PLCD1 and Caveolin-1 binding, and others are deficient for all of these properties.

**Conclusion:**

These observations highlight the importance of DLC1-START for the tumor suppressor function of DLC1 that is RhoGAP-independent. They also expand the versatility of START domains, as DLC1-START is the first found to bind PS, which promotes the binding to other proteins.

**Supplementary Information:**

The online version contains supplementary material available at 10.1186/s12943-021-01439-y.

## Background

*Deleted in liver cancer 1* (*DLC1*) is a tumor suppressor gene that was first discovered because it was deleted in hepatocarcinomas, and was later found to be downregulated, via genetic, epigenetic, and post-translational mechanisms, in many other tumor types, including colon/rectum, breast, prostate, and lung [1]. The downregulation of *DLC1* in tumors has been mostly attributed either to gene deletion or promoter DNA methylation, although other changes may also contribute to its decreased expression [2]. Very recently, a fine detailed analysis of several tumor types in the TCGA dataset revealed that tumor-associated point mutations in *DLC1* also occur frequently and can impair the biological functions of its encoded protein by several mechanisms [3].

*DLC1* encodes a multidomain protein that includes a Sterile alpha motif (SAM) at its N-terminus, a Rho GTPase activating protein (RhoGAP) domain, and a StAR-related lipid transfer (START) domain at its C-terminus. The RhoGAP domain negatively regulates Rho activity by promoting the conversion of active Rho-GTP to inactive Rho-GDP, which was initially hypothesized to be the sole basis of DLC1’s tumor suppressor functions, by remodeling the cytoskeleton and inhibiting neoplastic growth and cell migration [4].

However, DLC1 is also a scaffolding protein, and its interaction with other proteins, such as tensins, can contribute to DLC1 tumor suppressor activities in a RhoGAP-independent manner [5, 6], and reviewed in [7]. In addition, a GAP-dead DLC1 mutant in a non-small cell lung cancer cell line can still partially inhibit anchorage-independent growth [8], and an inactive form of DLC1 in NIH3T3 fibroblasts can partially inhibit cell migration [9]. Together, these findings lead to the conclusion that DLC1 exerts some of its tumor suppresive effects via GAP-independent mechanisms. Moreover, the activity of DLC1 can also be affected post-translationally, as several kinases, including PKA, AKT, and SRC, phosphorylate and ultimately affect DLC1 tumor suppressor activities [10, 11], and reviewed by [7].

In the current study, we have focused on the contribution of the DLC1 START domain (DLC1-START) to the full activity of DLC1, based on the hypothesis that the interaction of DLC1 with additional macromolecules through regions other than its RhoGAP domain may reveal new DLC1 scaffolding functions that could regulate its tumor suppressor activity, likely in a RhoGAP independent manner.

START domains are typically found in lipid transfer proteins, which can play diverse roles in several aspects of lipid biology, including lipid trafficking, lipid metabolism, and modulation of signaling events [12]. The identity of the lipids that bind each START domain is known for only a few members of the family, and thus far, no lipid has been found to bind the START domain of DLC1 or the other members of the DLC family, DLC2 and DLC3. Moreover, with the exception of the interaction of DLC1-START with Caveolin-1 [13], no other functional interactions have been linked to this domain. Therefore, the function of DLC1-START and its contribution to DLC1 functions remain largely uncharacterized.

Caveolin-1, the main component of caveolae [14], forms a functional complex with DLC1 in non-small cell lung cancer cells by interacting with a region whose binding is reduced by deletion of aminoacids 929 to 957 in DLC1-START. The interaction of DLC1 with Caveolin-1 was found to be essential for DLC1 to inhibit migration and anchorage-independent growth but not to affect the DLC1 RhoGAP activity [13], representing another example of a RhoGAP-independent mechanism of DLC1 tumor suppressor activities. Moreover, experimental analysis of The Cancer Genome Atlas database (TCGA) colorectal cancer-associated *DLC1* mutant (E966K), whose mutation is adjacent to the 929–957 deletion in DLC1-START, phenocopied these results, as it encoded a protein that was deficient in binding to Caveolin-1 in cells and displayed impaired DLC1 tumor suppressor activities (cell migration and anchorage-independent growth) without affecting its RhoGAP activity [3].

Phospholipase C delta 1 (PLCD1) was the first binding partner of DLC1 to be identified [15]. The PLC proteins have the ability to convert phosphatidylinositol bisphosphate (PIP2) to inositol trisphosphate (IP3) and diacylglycerol (DAG), which act both as scaffold and signaling molecules [16]. P122-RhoGAP, the rat homolog of DLC1, was found to interact with PLCD1, which was associated with an increase in the in vitro PIP2-hydrolyzing activity of PLCD1. In cells, this activity resulted in the release of actin-binding proteins, a process that accelerated the disassembly of actin fibers, and contributed, in conjunction with its RhoGAP activity, to morphological changes and detachment of adherent cells [15]. However, the precise region(s) of P122/DLC1 that interacts with PLCD1 has not been elucidated, although a C-terminal fragment that included the RhoGAP and START domains appeared to be important [15]. On the other hand, human DLC1 failed to activate the phospholipid hydrolysis activity of PLCD1 in vitro [8].

Here we have found that DLC1-START binds PLCD1 in addition to Caveolin-1. We also report the interaction of DLC1-START with a lipid, Phosphatidylserine (PS), that is crucial for the full activity of DLC1. Moreover, the binding of PS to DLC1 contributes to the interaction of DLC1 with PLCD1 and with Caveolin-1. In addition, we have identified several colorectal cancer-associated DLC1-START mutants that are deficient for all or some of these interactions and possess reduced tumor suppressor function but intact RhoGAP activity.

## Methods

### Reagents and antibodies

PS (DHPS, cat# L-3106) was purchased from Echelon and prepared as recommended by the supplier. Geneticin (G418) selection reagent was from Life Technologies. The source of the different primary antibodies used for western blot was as follows: mouse anti human DLC1 (BD, cat# 612021), mouse anti PLCD1 (A-4, Santa Cruz Biotechnologies, cat # sc-365,812), rabbit anti human Caveolin-1 (Abcam, cat# Ab2910), mouse anti GFP (clone B34, BioLegend, cat # 902601), mouse anti GST (B-14, Santa Cruz Biotechnologies, cat # sc-138), Rabbit anti C-terminal DLC1 (C2C3, GeneTex, cat # GTX113607), mouse anti Rho A, B, C (clone 55, EMD Millipore, cat # 05–778), mouse MBP-probe (R29.6, Santa Cruz Biotechnologies, cat # sc-13,564), and mouse anti Actin (clone AC-40, Sigma, cat # A4700). For Caveolin-1 or GFP immunoprecipitation, mouse anti Caveolin-1 (7C8, Novus Biologicals, cat # NB 100–615) or rabbit anti-GFP (Abcam, cat# Ab290) was used. For the other immunoprecipitations, the antibodies were the same as those used for western blotting.

### DNA constructs

*DLC1* variant 2 cDNA was used in this study for the generation of the different expression plasmids. pCDNA DLC1, pEGFP-DLC1, pEGFP DLC1 1–600, pEGFP DLC1 500–1091, pEGFP DLC1 1–110, pEGFP DLC1 1–492, pEGFP DLC1 80–400, pEGFP DLC1 400–500, pEGFP DLC1 500–798, pEGFP DLC1 609–848, pEGFP-DLC2, and pEGFP-DLC3 have already been described [5, 11, 13, 17]. To clone the DLC1-START domain containing region, the DLC1 sequence corresponding to amino acids 848–1091 was amplified by PCR using the primers listed in Supplementary Table 1 and the resulting PCR product was cloned into the pEGFP-C1 empty vector into EcoRI-SalI sites. GFP DLC1 825–1091 containing a stop codon at the end was cloned into EcoR1-Not1 site of pMTV5-HisA vector. The construct was synthesized by Genscript. MBP-DLC1 848–1091 was generated by PCR amplification of DLC1 848–1091 region using the primers listed in Supplementary Table 1 and cloning into the pMAL C2X vector into EcoRI-SalI sites. We cloned DLC1 848–1091 for expression in bacteria after attempts to express DLC1 START domain (878–1081) were unsuccessful because of solubility issues. pCDNA DLC1 848–1091 was subcloned from pEGFP DLC1 848–1091 by EcoRI-ApaI digestion. The GST-Caveolin-1 plasmid has already been described [13]. GST-DLC1 was generated by subcloning pCDNA DLC1 into pEBG vector using KpnI-NotI sites. GST PLCD1 (1–756), and GST PLCD1 1–140, 140–300, 300–450, 450–600, or 600–756 were cloned by PCR amplification of PLCD1 full length cDNA or corresponding fragments using primers listed in Supplementary Table 1 and the resulting PCR products were cloned into pEBG vector into KpnI-NotI sites. All plasmids were verified by sequencing. Lact-C2-GFP was a gift from Sergio Grinstein (Addgene plasmid # 22852).

### Mutagenesis

pEGFP-DLC1 929–957 Del, pEGFP-DLC1 R718A, and pEGFP-DLC1 E966K have already been described [3, 5, 13]. DLC1 point mutants were generated by Quick-Change Site Directed PCR mutagenesis kit following the manufacturer’s instructions (Stratagene), using pEGFP-DLC1 or pEGF-DLC1 848–1091 plasmids as templates. The primers used are listed in Supplementary Table 1.

The pEGFP-Lact-C2 3A mutant (W26A,W33A,F34A) was also generated with the use of Quick-Change Site Directed PCR mutagenesis kit, using the primers described elsewhere [18]. All plasmids were verified by sequencing.

### Cell culture, transfections, and generation of stable cell lines

H1703, H358 and H1299 NSCLC cell lines were grown in RPMI media supplemented with 10% FBS. 293 T and 293H cells were grown in DMEM media supplemented with 10% FBS. CHO-K1 cells were maintained in Ham’s F-12 medium supplemented with 10% newborn calf serum. PSA3 and PSB2 cells were gifts from Dr. Osamu Kuge (Kyushu University) and Dr. Gregory Fairn (University of Toronto), respectively, and were routinely maintained in Ham’s F-12 supplemented with 10% newborn calf serum and 30 μM PS (from bovine brain, Sigma) liposomes prepared as described [19]. For experiments designed to check the effect of PS deficiency, PSA3 and PSB2 cells were cultured for 3 to 5 days in the absence of PS. Insect S2 cells were grown in Schneider’s medium containing 10% FBS. CRL-1739 cells were grown in F-12 K medium supplemented with 10% FBS. MKN-28 cells were maintained in RPMI media supplemented with 10% FBS. All cells were maintained in a humidified atmosphere containing 5% CO_2_.

Transient transfections were done using Lipofectamine 3000 (Invitrogen), following the manufacturer’s protocol, except for 293 T or 293H cells, which were routinely transfected using Lipofectamine 2000 (Invitrogen), according to the manufacturer’s protocol, and cultured for 48 h before samples were processed. For PS sequestration experiments, cells were transfected with plasmids expressing the C2 domain of Lactadherin WT, or 3A mutant, followed by a second transfection with DLC1 or DLC1-START domain containing plasmids 24 h later. Cells were processed 24–36 h after the second round of transfection. For the generation of stable clones, transfected H358 cells were cultured for several weeks in media containing 0.8 μg/ml G418 selection.

### Bacterial expression of MBP proteins

BL21 DE3 pLysS *E. coli* chemically competent cells (Invitrogen) were transformed with pMAL C2X empty vector or pMAL C2X DLC1 848–1091. Cultures containing the appropriate selection antibiotics and 0.2% glucose were grown at 37 °C until reaching an OD of about 0.6 and induced with 0.2 mM IPTG for about 4 h. Bacterial cultures were harvested, and pellets were resuspended in column buffer (20 mM Tris-HCl, 200 mM NaCl, 1 mM EDTA) containing Complete protease inhibitors (Roche). The suspension was then sonicated 20–30 s at a time for about 3–4 min with about 15 s on ice between each burst of sonication, and the lysate centrifuged for 20 min at 30,000 rpm at 4 °C. Purification of MBP proteins was performed with amylose resin (New England Biolabs, check) overnight at 4 °C. The resin was washed 3 times with column buffer, and the purity and amount of bound MBP proteins was checked by SDS-PAGE and Coomassie blue staining. For in vitro binding experiments to PS beads, MBP proteins were eluted from the amylose resin by overnight incubation at 4 °C in column buffer containing 10 mM maltose (Sigma-Aldrich).

### MBP and GST pull-down assays

Total (whole cell) lysates from transfected cells were obtained by solubilizing cells with 1X RIPA buffer (EMD Millipore; 0.05 M Tris-HCl pH 7.4, 0.15 mM NaCl, 0.25% deoxycholic acid, 1% NP-40, 1 mM EDTA) containing protease and phosphatase inhibitors. Lysates were clarified by centrifugation at 14,000 rpm for 10 min at 4 °C, and protein was quantified using a BCA kit (Thermo Fisher Scientific), following manufacturer’s instructions. For GST pull-down assays, total lysates were prepared from cells co-transfected for 48 h with GST or GST fusion plasmids along with GFP or GFP DLC1 wild type or mutant constructs. Pull-downs were performed by incubating equal amounts of whole cell protein extracts with MBP or MBP DLC1 848–1091 purified proteins for MBP pull-down assays, or with glutathione Sepharose-4B beads (GE Healthcare) for GST-pull down assays, and rotating the samples for 4 h at 4 °C. When indicated, 100 μM PS (Echelon) was added to the binding reaction. Pellets were washed three times with 1X RIPA buffer, boiled in loading buffer, and proteins were separated by SDS PAGE, followed by immunoblotting with the corresponding antibodies. A portion of the cell extracts was used as loading control to verify the expression of the transfected proteins.

### Immunoprecipitation and Western blotting

Total lysates were prepared as described above. For immunoprecipitations, equal amounts of proteins were pre-cleared with 15–20 μl TrueBlot anti-Rabbit or anti-Mouse Ig IP agarose beads (Rockland) and incubated with the indicated antibodies for 1 h at RT. After incubation, 30 μl of TrueBlot agarose beads was added to each immune reaction and rotated at 4 °C overnight. The immunopellets were then washed 3 times with 1X RIPA buffer and boiled in 40 μl loading buffer. Proteins were separated on NuPage 4–12% Bis Tris gels (Thermo Fisher scientific), transferred to nitrocellulose membranes, and analyzed by immunoblotting using specific primary antibodies, followed by secondary anti-IgG antibodies conjugated with HRP (GE Healthcare). For immunoprecipitations, mouse or rabbit Trueblot Ig HRP secondary antibodies (Rockland) were used. Immunoreactive bands were detected by chemiluminescence using KwikQuant Western Blot Detection kit and Kwikquant Imager acquisition system (Kindle Biosciences).

### Fractionation studies

Transfected cells were briefly tripsinized, and the resulting pellets were processed for the preparation of membrane and cytosolic protein lysates, using a Mem-PER Plus Membrane Protein Extraction Kit (Thermo Fisher Scientific), following the manufacturer’s instructions. For the preparation of total lysates, an aliquot from the same samples was solubilized in 1X RIPA buffer, as described above. The resulting total, cytosolic and membrane lysates were quantified using a BCA kit (Thermo Fisher Scientific), and equal amounts of each fraction were loaded onto Nupage 4–12% Bis Tris gels (Thermo Fisher Scientific), transferred onto nitrocellulose membranes, and analyzed by immunoblotting using specific antibodies.

### Rho activation assay

Rho Assay Reagent (Rhotekin RBD) (EMD Millipore) was used to measure active GTP-bound RhoA, following the manufacturer’s protocol. Briefly, cells were lysed in Mg2+ lysis buffer (MLB), and the total protein concentration was estimated by BCA assay (Thermo Fisher Scientific). Equal amounts of protein were incubated with Rhotekin-RBD beads for 2 h at 4 °C, and beads were washed three times with MLB buffer. Samples were then subjected to 4–12% SDS-PAGE and transferred onto nitrocellulose membranes (Thermo Fisher Scientific). The amount of GTP-RhoA was detected by immunoblotting, using RhoA antibody (EMD Millipore).

### Lipid pull-down assays

Protein lysates were prepared by solubilizing cells in 1X RIPA buffer (EMD Millipore). Prior to adding the protein lysate, phosphatidylserine (PS) or control beads (Echelon) were incubated with Perfect Block (Boca-Scientific) as a blocking reagent for 1 h at RT. After incubation, beads were washed three times with 1X RIPA buffer, and then pull-downs were performed by incubating equal amount of proteins with the pre-blocked PS or control beads for 4 h at 4 °C, followed by extensive washing with 1X RIPA buffer (3–4 times). Proteins were separated by SDS-PAGE, transferred to nitrocellulose membranes, and the lipid-bound proteins were detected by immunoblotting with specific antibodies.

### Lipid overlay assays

Cells were transfected with individual constructs, and 48 h later the cells were harvested and lysed. Cell extracts containing 100 micrograms of protein were used per overlay assay. For preliminary binding, commercial membrane lipid strips (Echelon Biosciences Inc., P-6002) were used. For PS lipid overlay assays, PS (Avanti polar lipids, PS-840032) was prepared as 1 mM stock solution, and different quantities of the lipid were spotted on Hybond-C extra nitrocellulose membranes (Amersham Biosciences). The membranes were incubated with the cell extracts overnight, blocked, and immunoblotted with specific antibodies. All steps were carried out in the presence of 3% BSA.

### Cell proliferation, cell migration and soft agar cell growth assays

For proliferation assays, 24 h after transfection, 0.5X10^4^ cells were plated in 6-well plates in triplicate, and cells were quantified in a cell counter (Nexcelom) at the indicated times. The transwell cell migration assays were performed in duplicate, using 6.5 mm diameter Falcon cell culture inserts (8 mM pore size, Falcon) in 24 well cell culture plates. 1X10^5^ cells in serum-free media were transferred to the upper chamber, while the lower chamber contained media with 10% FBS. Following overnight incubation, the cells remaining on the upper surface were removed with a cotton swab. Migrated cells were fixed, visualized microscopically, photographed, and quantified using Image J. For soft agar assays, 1X10^5^ stably transfected cells were mixed with complete media containing 0.4% of ultrapure agar (Invitrogen) and placed over 0.6% basal agar in 60 mm dishes. 3 to 5 plates per transfectant were used. Cells were grown for 3–4 weeks, and colonies were stained with Nitrotetrazolium Blue Chloride (1 mg/ml), photographed by microscopy, and quantified with a colony counter.

### Homology modeling of the DLC1-START domain and structural analysis

The homology model of the START domain (887–1091) was generated using comparative protein modeling implemented in the program suite incorporated in SWISS-MODEL (http://swissmodel.expasy.org) [20]. To generate a model of DLC1 START domain, crystal structures of the human DLC2-START domain (PDB ID: 2PSO) and CERT-START domain (PDB ID: 2E3O) that shares 57 and 17% amino acid sequence identity, respectively, with the DLC1-START domain, were used as templates [21, 22]. Evaluation and quality estimation of homology models obtained using these two templates showed a high degree of similarity except for the initial N-terminal helix, which points away from the core of the protein in the DLC2-START domain due to crystal packing interaction. In all other START domain structures that have been solved so far, this N-terminal helix interacts with and stabilizes the central beta-barrel. For this reason, the N-terminal helix was modelled using the CERT-START domain as the template. The final structure was selected based on the value of the Global Model Quality Estimation (GMQE). A higher GMQE value of 0.77 (varies between 0 and 1) reflects the better accuracy of the modeled structure.

To identify the lipid-binding site in the DLC1-START domain, we carried out cavity analysis with the program CavityPlus [23], which uses a structural geometry-based method for ligand-binding site detection and analysis. The orientation of the DLC1-START domain at the membrane was analyzed using the orientation of proteins in membranes (OPM) computational approach [24]. In OPM analyses, optimal rotational and translational positioning of the protein to the lipid bilayer is achieved by minimizing protein transfer energy from water to the membrane hydrocarbon core. Structural figures were generated using PyMOL (Schrödinger, LLC).

### Identification of missense mutations of DLC1 START domain from TCGA dataset

The DLC1 mutations selected from this study are from two sources: Catalog of Somatic Mutations in Cancer (COSMIC) database [COSMIC dataset (Cosmic v74, 2015)] and TCGA harmonized mutation data from NCI Genomic Data Commons (GDC) Data Portal. DLC1 sequence from both sources is isoform 1 (NP_872584), which is 437 amino acid residues longer than sequence from current study (isoform 2, NP_006085).

### Protein correlation analysis

The processed proteomics datasets and clinical information from The National Cancer Institute’s Clinical Proteomic Tumor Analysis Consortium (CPTAC, https://proteomics.cancer.gov/programs/cptac) were used for the data analysis. Briefly, the data of lung adenocarcinoma and lung squamous cell carcinoma were downloaded directly from CPTAC portal and load into mysql table (mysql community server 8.0.17). A PHP/mysql based program was used for generating plots to compare DLC1, CAV1 and PLCD1 protein abundance between tumor and adjacent normal and performing correlation analysis. *P* < 0.05 is considered statistically significant.

## Results

### The DLC1 START domain binds phospholipase C delta 1 (PLCD1) in addition to Caveolin-1

**Fig. 1:**
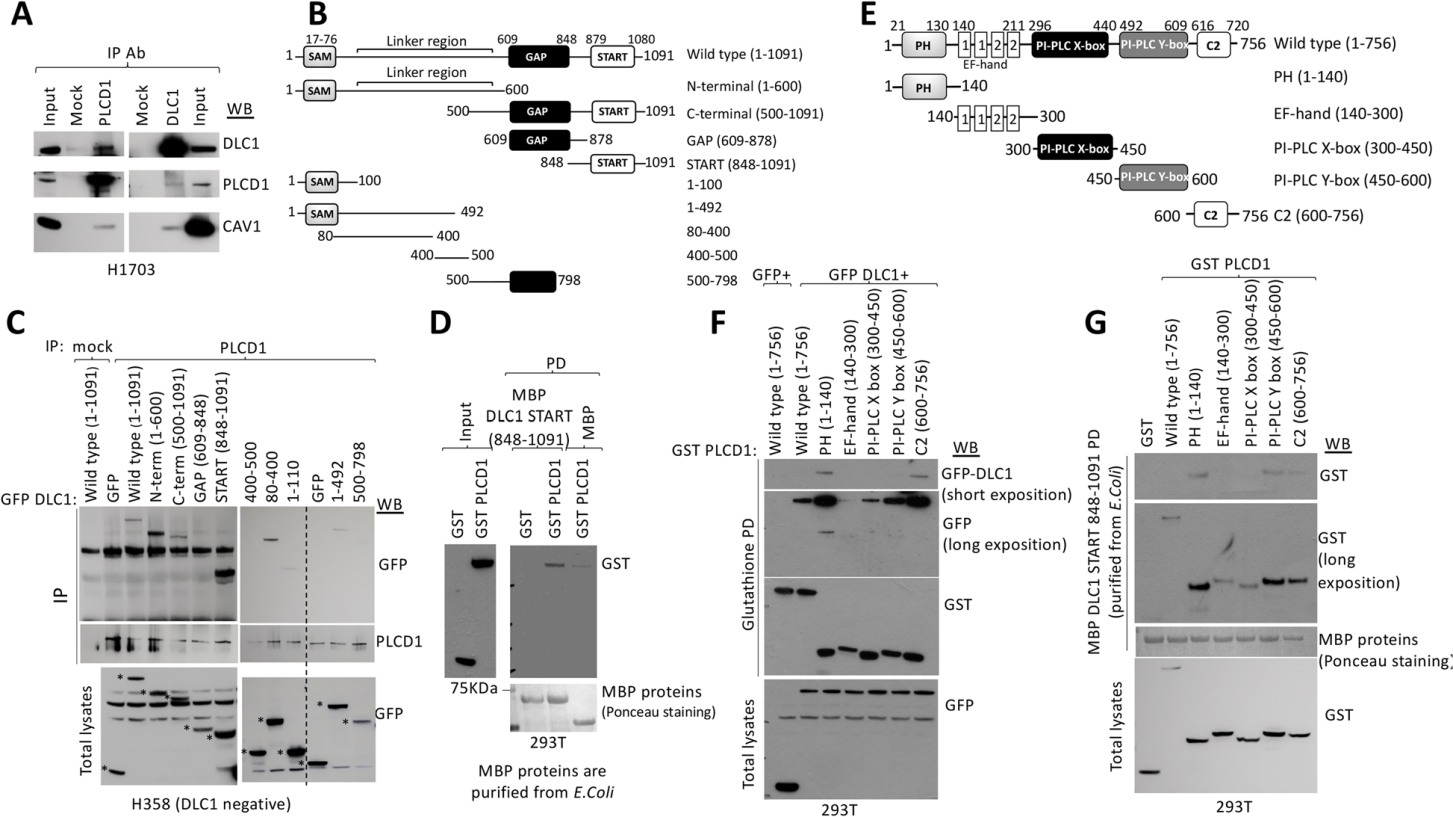
Mapping of DLC1 and PLCD1 interacting regions. **A** Co-immunoprecipitation of endogenous PLCD1, DLC1 and Caveolin-1 proteins in H1703 cells. The indicated antibodies for immunoprecipitation (IP) and Western blot (WB) were used. Input represents a fraction of the H1703 cell lysate before immunoprecipitation. **B** Schematic representation of the domain organization of DLC1 (top) and DLC1 constructs cloned as GFP-fusion proteins for immunoprecipitation in C. **C** PLCD1 Immunoprecipitation (IP) in cell lysates from H358 cells overexpressing control (GFP) or GFP-fusion plasmids spanning several regions in DLC1 followed by Western blot (WB) using the indicated antibodies. The expression of all GFP-fusion plasmids in total lysates is shown in the bottom panel. Asterisks (*) indicate the expression of the corresponding GFP-fusion proteins. **D** MBP pull-down (PD) in 293 T cells expressing GST control or GST PLCD1 using MBP control or MBP-DLC1 START 848–1091 purified proteins (from E.Coli), followed by GST Western blot (WB). The amount of MBP-purified proteins is shown as stained with Ponceau S (bottom panel). Input represents a fraction of the 293 T cell lysates before pull-down. **E** Schematic representation of the domain organization of PLCD1 (top) and PLCD1 constructs cloned as GST-fusion proteins to be used in F and G. **F** Glutathione pull-down (PD) in 293 T cells transfected with GST-PLCD1 plasmids encompassing several PLCD1 domains and GFP or GFP DLC1, followed by GFP and GST western blot. The expression of all GFP-fusion plasmids in total lysates is shown (bottom). **G** Pull-down (PD) using MBP DLC1 START 848–1091(purified from *E. coli*) and GST or GST-PLCD1 domains, followed by GST western blot. The amount of MBP- purified protein is shown as stained with Ponceau S. Expression of GST fusion proteins was checked by GST western blot in total lysates (bottom)

As reported for the rat DLC1 homolog, immunoprecipitation studies confirmed complex formation between PLCD1 and DLC1 [15] in H1703 cells, a human lung cancer cell line that expresses readily detectable levels of both proteins, as well as between PLCD1 and Caveolin-1 (Fig. 1A). To map the region(s) of the 1091 amino acid human DLC1 protein (isoform 2) that interact with PLCD1, plasmids encoding different regions of DLC1, representing N-terminal, C-terminal, START, and GAP domains, were cloned as GFP-fusion proteins (Fig. 1B) and expressed in H358 cells, which do not express DLC1 protein, and endogenous PLCD1 was immunoprecipitated from cell extracts. Figure 1C shows that PLCD1 was able to interact with two non-overlapping regions of DLC1: a DLC1 N-terminal region (aminoacids 1–600) and a C-terminal region (amino acids 848–1091) that includes the DLC1-START domain (amino acids 878–1081). Under the same conditions, the GAP domain (609–848) did not immunoprecipitate with PLCD1. Further mapping indicated that within the N-terminal (1–600) region, PLCD1 bound DLC1 amino acids 80–400 (Fig. 1C). Moreover, MBP pull-down experiments using the DLC1 START-containing domain (amino acids 848–1091) purified from *E. coli* was able to recapitulate the interaction of this domain with PLCD1 in extracts from 293 T cells that expressed PLCD1 (Fig. 1D), demonstrating that isolated DLC1-START produced in bacteria was sufficient for PLCD1 interaction.

The full-length 756 amino acid PLCD1 protein contains several conserved domains, including Pleckstrin homology (PH), EF-hand, catalytic X and Y, and C2 domains [25] (Fig. 1E). To delineate more precisely the regions of PLCD1 that interact with DLC1, we cloned as GST fusion plasmids five individual fragments of PLCD1 that each encoded about 150 PLCD1 amino acids, which approximately correspond to each of the above domains (Fig. 1E). The GST-fusion plasmids were co-transfected along with GFP-DLC1 into 293 T cells, and Glutathione pull-down experiments were performed (Fig. 1F). The regions containing the PH and C2 domains, which span PLCD1 aminoacids 1–140 and 600–756, respectively, bound preferentially to full-length DLC1. Similar results were obtained when extracts from 293 T cells expressing one of the PLCD1-fragments were subjected to in vitro pull-down with MBP-DLC1 848–1091 (START-domain containing region) purified from bacteria, as PLCD1 regions 1–140 and 450–756 bound strongly to DLC1-START (Fig. 1G).

### Caveolin-1 and PLCD1 cooperate with DLC1 to inhibit migration, but compete with each other for DLC1 binding

**Fig. 2:**
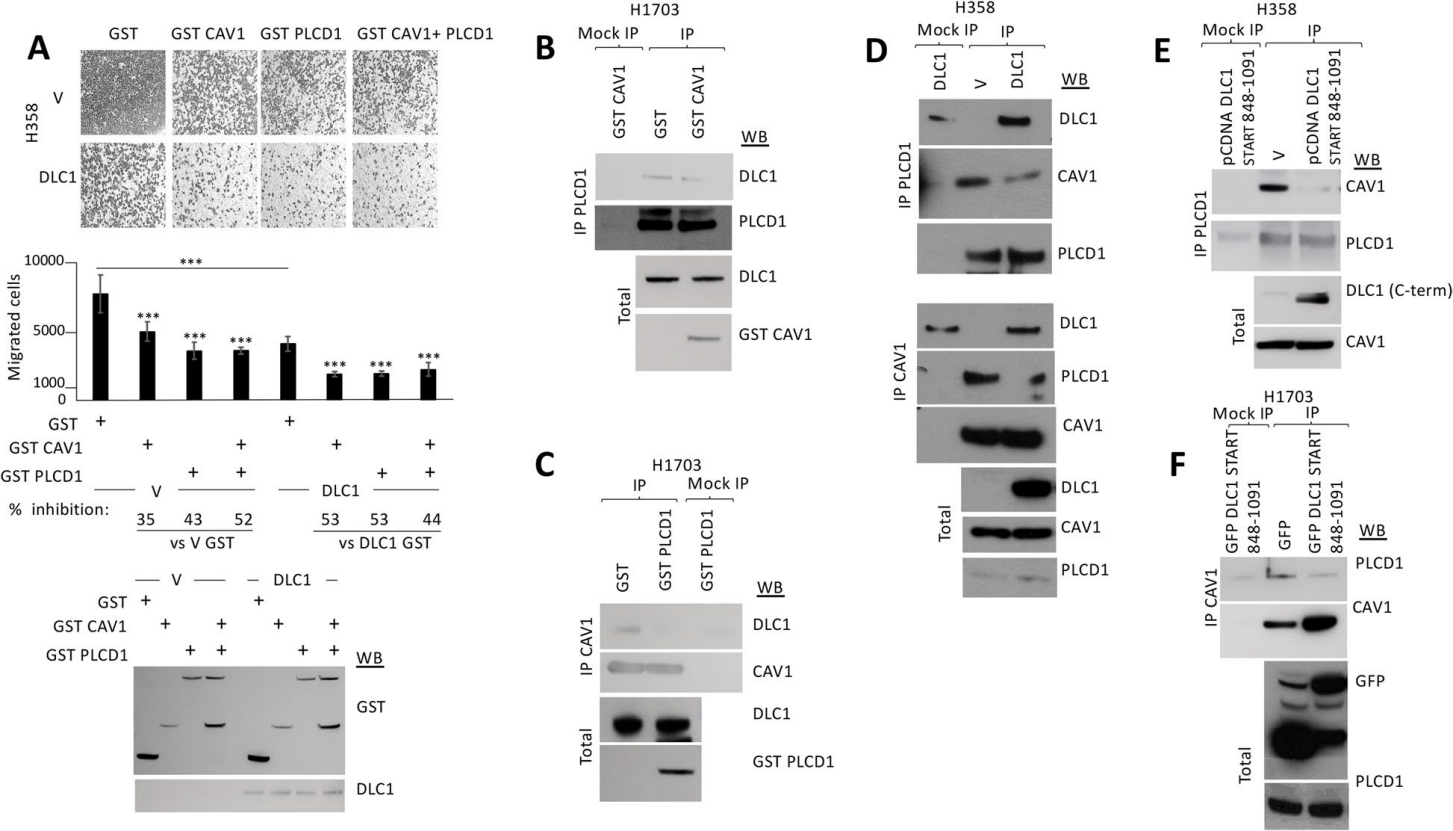
Caveolin-1 and PLCD1 cooperate with DLC1 to inhibit cell migration but their binding to DLC1 is mutually exclusive. **A** Migration in transwells of stable H358 control (V) or DLC1-overexpressing cells transiently transfected with GST control, GST Caveolin-1 (GST CAV1), GST PLCD1 or GST CAV1 + GST PLCD1. Migrated cells were fixed and photographed (top panel shows a representative image from each condition). The number of migrated cells were quantified using Image J (middle panel), and the percentage (%) of inhibition was calculated from a total of four random images taken from each experiment duplicate. Bars represent mean +/− SD. t-test was performed for statistical analysis (***:*p* < 0.005). The correct expression of GST proteins and DLC1 was checked by Western blot (WB). **B** PLCD1 Immunoprecipitation in H1703 cells transfected with GST control or GST Caveolin-1 (GST CAV1) plasmids, followed by Western blot (WB) using the indicated antibodies. **C** Caveolin-1 (CAV1) Immunoprecipitation (IP) in H1703 cells transfected with GST control or GST PLCD1 plasmids, followed by Western blot (WB) using the indicated antibodies. The correct expression of GST fusion proteins in B and C is shown from total lysates (bottom panels). **D** Immunoprecipitation (IP) using PLCD1 (top) or Caveolin-1 (CAV1, middle) antibodies in H358 cells transfected with empty vector (V) or pCDNA DLC1, followed by Western blot (WB) using the indicated antibodies. Correct expression of proteins in total lysates is shown in the bottom panel. **E** PLCD1 Immunoprecipitation (IP) in H358 cells transfected with empty vector (V) or a plasmid encoding the DLC1 START domain-containing region (DLC1 848–1091), followed by Western blot (WB) using the indicated antibodies. The correct expression of all proteins in total lysates is shown (bottom panel). **F** Caveolin-1 Immunoprecipitation (IP) in H1703 cells transfected with GFP or GFP-DLC1 START domain containing region (848–1091), followed by Western blot (WB) using the indicated antibodies. The correct expression of proteins in total lysates is shown (bottom panel)

We next sought to assess the impact of PLCD1 on the ability of DLC1 to inhibit migration. H358 control or DLC1-expressing stable clones were transiently transfected with PLCD1, and the cells were allowed to migrate on transwells. PLCD1 inhibited migration in control cells and cooperated with DLC1 to further inhibit migration in the DLC1-expressing cells (Fig. 2A). The same effect was also observed when Caveolin-1 was overexpressed (Fig. 2A), confirming, as previously reported [13], that Caveolin-1 contributes to the tumor suppressor activity of DLC1. In contrast to the observed cooperation when DLC1 was co-expressed with either PLCD1 or Caveolin-1, co-expression of PLCD1 and Caveolin-1 together did not result in additional inhibition of migration, whether expressed in control H358 cells or the DLC1-expressing cells (Fig. 2A), suggesting that both proteins may occupy the same signaling pathway, at least for cell migration.

These observations suggested that DLC1, Caveolin-1 and PLCD1 may be coregulated in cancer, as previously seen for DLC1 and Caveolin-1 in NSCLC [13]. We therefore analyzed the CPTAC protein dataset for DLC1, Caveolin-1 and PLCD1 expression in lung adenocarcinoma (Supplementary Figure 1a) and lung squamous cell carcinoma (Supplementary Figure 1b) compared to adjacent normal lung. The results confirmed DLC1, Caveolin-1 and PLCD1 protein levels were lower in tumor tissue compared to normal adjacent lung. In addition to confirming the previously identified association between DLC1 and Caveolin-1, which had been demonstrated with a different dataset, there was also a positive correlation between DLC1 and PLCD1 expression in both lung tumor types, as well as between Caveolin-1 and PLCD1.

Since both PLCD1 and Caveolin-1 bind DLC1, we investigated whether there might be competition between these two proteins for binding DLC1, which could be at least a partial explanation for not observing cooperative inhibition of migration when all three proteins were co-expressed. In immunoprecipitation studies, overexpression of Caveolin-1 in H1703 cells displaced the endogenous interaction between DLC1 and PLCD1, and likewise, the overexpression of PLCD1 in the same cells displaced the Caveolin-1 to DLC1 interaction (Fig. 2B and C, respectively). Furthermore, overexpression of DLC1 in H358 DLC1-negative cells reduced complex formation between Caveolin-1 and PLCD1 in immunoprecipitation assays (Fig. 2D). Similarly, overexpression of the START domain-containing region alone (848–1091) was also able to effectively displace the interaction between endogenous Caveolin-1 and PLCD1 (Fig. 2E and F) in two different cell lines. Taken together, these results imply that each DLC1 START domain only binds one of the proteins (Caveolin-1 or PLCD1) at a time, and competition between these two proteins for DLC1-START binding may occur.

### DLC1-START binds Phosphatidylserine (PS) in addition to Caveolin-1 and PLCD1

**Fig. 3:**
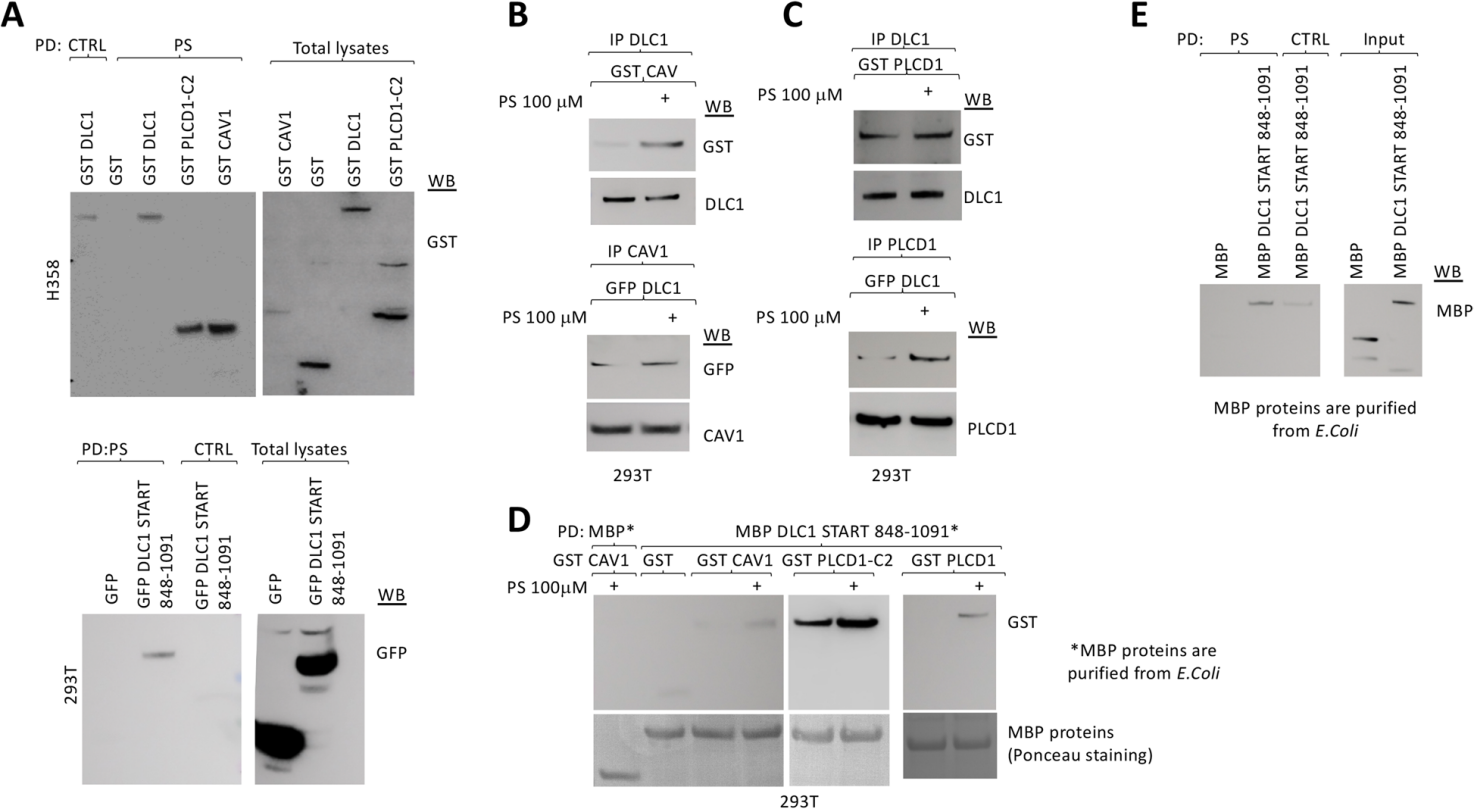
DLC1-START binds Phosphatidylserine and this binding improves its binding to Caveolin-1 or PLCD1. **A** Pull-down (PD) using control (ctrl) or PS-coated beads in H358 (top) or 293 T (bottom) cells overexpressing the indicated GST or GFP-fusion plasmids, followed by GST or GFP Western blot (WB). The correct expression of all plasmids was verified in total lysates by WB. **B** Immunoprecipitation (IP) in 293 T overexpressing GST Caveolin-1 (GST CAV) (top) or GFP DLC1 (bottom) using the indicated antibodies, in the presence or absence of 100 μM PS added to the binding reaction. Immunocomplexes were analyzed by Western blot (WB) using the indicated antibodies. **C** Immunoprecipitation (IP) in 293 T overexpressing GST PLCD1 (GST PLCD1) (top) or GFP DLC1 (bottom) using the indicated antibodies, in the presence or absence of 100 μM PS added to the binding reaction. Immunocomplexes were analyzed by Western blot (WB) using the indicated antibodies. **D** Pull-down (PD) using MBP control or MBP DLC1 START 848–1091 beads in 293 T overexpressing the indicated GST-fusion plasmids, in the presence or absence of 100 μM PS added to the binding reaction, followed by GST Western blot. The amount of MBP-purified proteins is shown as stained with Ponceau S (bottom panel). **E** Pull-down (PD) using control (ctrl) or PS-coated beads and MBP control or MBP DLC1 START 848–1091 purified proteins (eluted from amylose resin in the presence of maltose), followed by MBP Western blot (WB). The purity and amount of MBP-purified proteins is shown in the western blot from the inputs, which represents a fraction of the total purified protein used in the pull-down

START domains in other proteins bind lipids [12], but no lipid has yet been identified to bind to DLC1-START. We surveyed the binding of DLC1-START to several lipids. Preliminary lipid overlay assays using 293 T cell extracts overexpressing GFP-DLC1 848–1091 and commercial lipid strips showed that DLC1-START may bind cardiolipin, phosphatidic acid, and PS (Supplementary Figure 2A). We further demonstrated the interaction of PS with full-length DLC1 or DLC1-START by pull-down, using lipid-coated beads and protein lysates expressing the different protein(s) of interest (Fig. 3A). Full-length DLC1 was able to specifically bind PS in cells (top panel), and DLC1-START was sufficient for this interaction (bottom panel). As previously reported, this assay also detected an interaction between PS and the C2 domain of PLCD1 [25, 26], as well as between PS and Caveolin-1 [27] (Fig. 3A, top panel). In an additional approach for evaluating the binding of DLC1-START to PS, we performed a lipid overlay assay, using different concentrations of PS in two different cell lines (293 T and insect S2) that overexpressed DLC1-START. Using this assay, DLC1-START was able to efficiently and specifically bind PS (Supplementary Figure 2B). The lipid overlay assay showed that DLC2 and DLC3 proteins also interacted with PS (Supplementary figure 2C), suggesting that PS binding is a general property of the three DLC proteins.

### PS increases binding between full-length DLC1 or DLC1-START and Caveolin-1 or PLCD1

Since we detected complex formation between DLC1 and Caveolin-1 or PLCD1, and all three proteins appeared to bind PS, we investigated whether the presence of PS might affect the protein-protein interactions in the in vivo extracts. The in vitro addition of exogenous PS (100 μM) to the immunoprecipitation reaction increased the interaction between DLC1 and Caveolin-1 (Fig. 3B), as well as that between DLC1 and PLCD1 (Fig. 3C). Furthermore, pull-downs using bacterially purified DLC1-START in the presence or absence of PS and protein lysates expressing Caveolin-1 or PLCD1 (full-length or its C2 domain) also indicated that PS increased the interaction between DLC1-START and Caveolin-1 or PLCD1 (Fig. 3D).

To address whether DLC1 binding to PS was mediated by PLCD1 and/or Caveolin-1, we used extracts from CRL-1739 cells, which express DLC1 but do not express Caveolin-1 or PLCD1 (Supplementary figure 3A), to evaluate the ability of endogenous DLC1 to bind PS-coated beads (Supplementary figure 3B). In the absence of Caveolin-1 and PLCD1 (GST-transfected control cells), DLC1 was able to bind to PS, and this binding was not significantly altered when Caveolin-1 or PLCD1 were expressed exogenously, alone or in combination (Supplementary figure 3B). In vitro pull-down experiments, using PS-coated beads and MBP or MBP DLC1 848–1091 proteins purified from *E. coli*, confirmed direct interation between DLC1-START and PS (Fig. 3E). These results indicated that the binding of PS to DLC1 can occur independently of the presence of Caveolin-1 and/or PLCD1. We next wanted to confirm that the binding of Caveolin-1 or PLCD1 to DLC1-START could occur in the absence of the other protein, and determine whether the interactions were influenced by the in vitro addition of PS. As determined by pull-downs +/− PS using bacterially purified DLC1-START and Caveolin-1 or the C2 domain of PLCD1 expressed in CRL-1739 cells, the PS-induced increased interaction between DLC1-START and Caveolin-1, and between DLC1-START and PLCD1 can occur when only two of the three proteins were co-expressed (Supplementary figure 3C). Taken together, these results suggest PS is a plausible physiologic interacting lipid of DLC1-START, whose binding may promote binding to other interacting partners through this domain.

### PS is important for DLC1 function

**Fig. 4:**
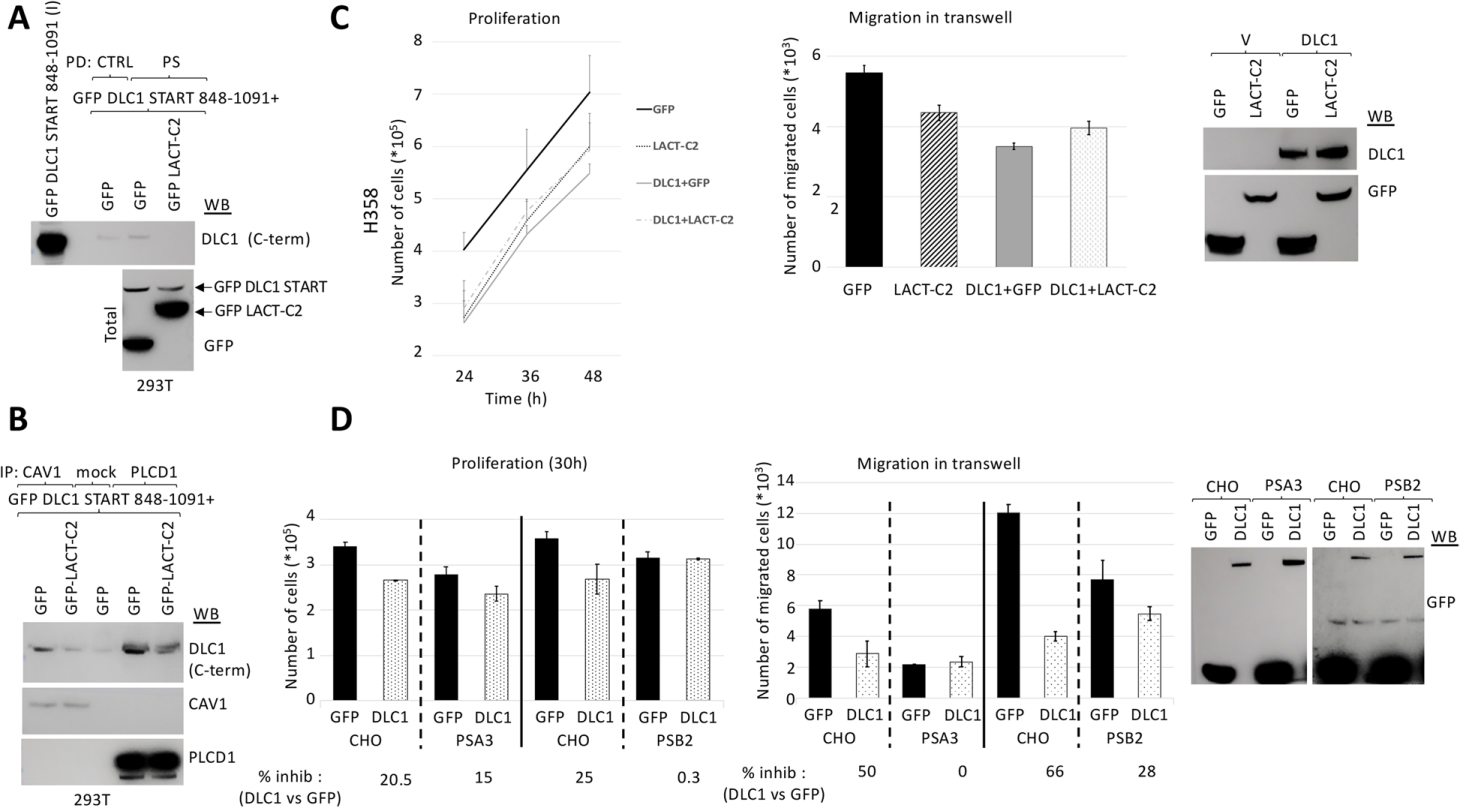
PS is essential for DLC1 inhibition of proliferation and migration. **A** Pull-down (PD) using control (ctrl) or PS-coated beads in 293 T overexpressing GFP DLC1 START 848–1091 and GFP or the C2 domain of Lactadherin (GFP-LACT-C2), followed by Western blot (WB) using an antibody that recognizes the C-terminal domain of DLC1 (DLC1 C-term). Input (I) represents a fraction of the protein lysates before pull-down. The correct expression of all plasmids in total lysates was checked by GFP Western blot (bottom panel). **B** Caveolin-1 or PLCD1 Immunoprecipitation (IP) in same cell lysates from A, followed by Western blot (WB) with the indicated antibodies. **C** Stable H358 control or DLC1-overexpressing cells were transfected with GFP or the C2 domain of Lactadherin (LACT-C2). 24 h after transfection, cells were plated at the same density and allow to proliferate for the indicated times (left panel) and counted, or to migrate in transwells overnight (middle panel). Migrated cells were fixed and photographed, and the number of migrated cells was quantified using Image J, from a total of four images taken from each experiment duplicate. The correct expression of all plasmids was checked by Western blot (WB) (right panel). **D** Parental CHO or PS-deficient mutant cell lines (PSA3 and PSB2) were transfected with GFP control or GFP-DLC1. 24 h after transfection, all cells were plated at the same density and allow to proliferate for 30 h (left panel) and counted, or to migrate in transwells overnight (middle panel). Migrated cells were fixed and photographed, and the number of migrated cells was quantified using Image J, from a total of four images taken from each experiment duplicate. Bars represent mean +/− SD. The percentage (%) of inhibition was calculated for DLC1 transfected cells compared to GFP control transfected cells, for each cell line. The correct expression of all plasmids was checked by GFP Western blot (WB) (right panel)

To examine the effect of PS on DLC1 functions, we sought to block the binding of DLC1 with PS by making use of the C2 domain of Lactadherin, a protein that binds PS with high affinity and prevents its interaction with other lipid-binding proteins [18]. The presence of Lactadherin-C2 in the cell efficiently prevented the binding of the START domain of DLC1 to PS-coated beads (Fig. 4A) in PS pull-down studies, and also to Caveolin-1 and PLCD1 in immunoprecipitation assays (Fig. 4B). Mutation of key residues in Lactadherin-C2 that prevents its binding to PS and renders the protein cytosolic [18] restored formation of complexes between DLC1-START and Caveolin-1 or PLCD1 (Supplementary Fig. 4A). The presence of Lactadherin-C2 also reduced the binding of full-length DLC1 to Caveolin-1 and PLCD1(supplementary Fig. 4B).

To investigate whether blocking the interaction of DLC1 with PS affected biological functions of DLC1, we performed proliferation and migration assays on transwell plates in H358 DLC1-expressing stable clones that were transiently transfected with the C2-domain of Lactadherin (Fig. 4C). The results showed that Lactadherin-C2 reduced the ability of DLC1 to inhibit proliferation (Fig. 4C, left panel) and migration (Fig. 4C, middle panel). In a different cell system, Chinese hamster ovary cells (CHO), we took a genetic approach to investigate the role of PS, as PS is synthesized in the ER by two separate enzymes, PS synthase (PSS) 1 and 2 in mammalian cells. We used two mutant CHO cell lines, one with reduced PSS 1 (PSA3) and the other with reduced PSS 2 (PSB2) activities, each of which leads to cells with lower PS content than wild type cells [28, 29]. When DLC1 was transfected in CHO wild type and mutant cells, the inhibition of proliferation (Fig. 4D, left panel) and migration (Fig. 4D, middle panel) by DLC1 was attenuated in the mutant cells with low PS (PSA3 and PSB2) compared to parental cells (Fig. 4D). These results strongly suggest that the binding of DLC1 to PS in the cell is essential for DLC1 functionality.

### Cancer-associated DLC1-START mutants have reduced DLC1 inhibition of migration and anchorage-independent growth but retain DLC1 RhoGAP activity

**Fig. 5:**
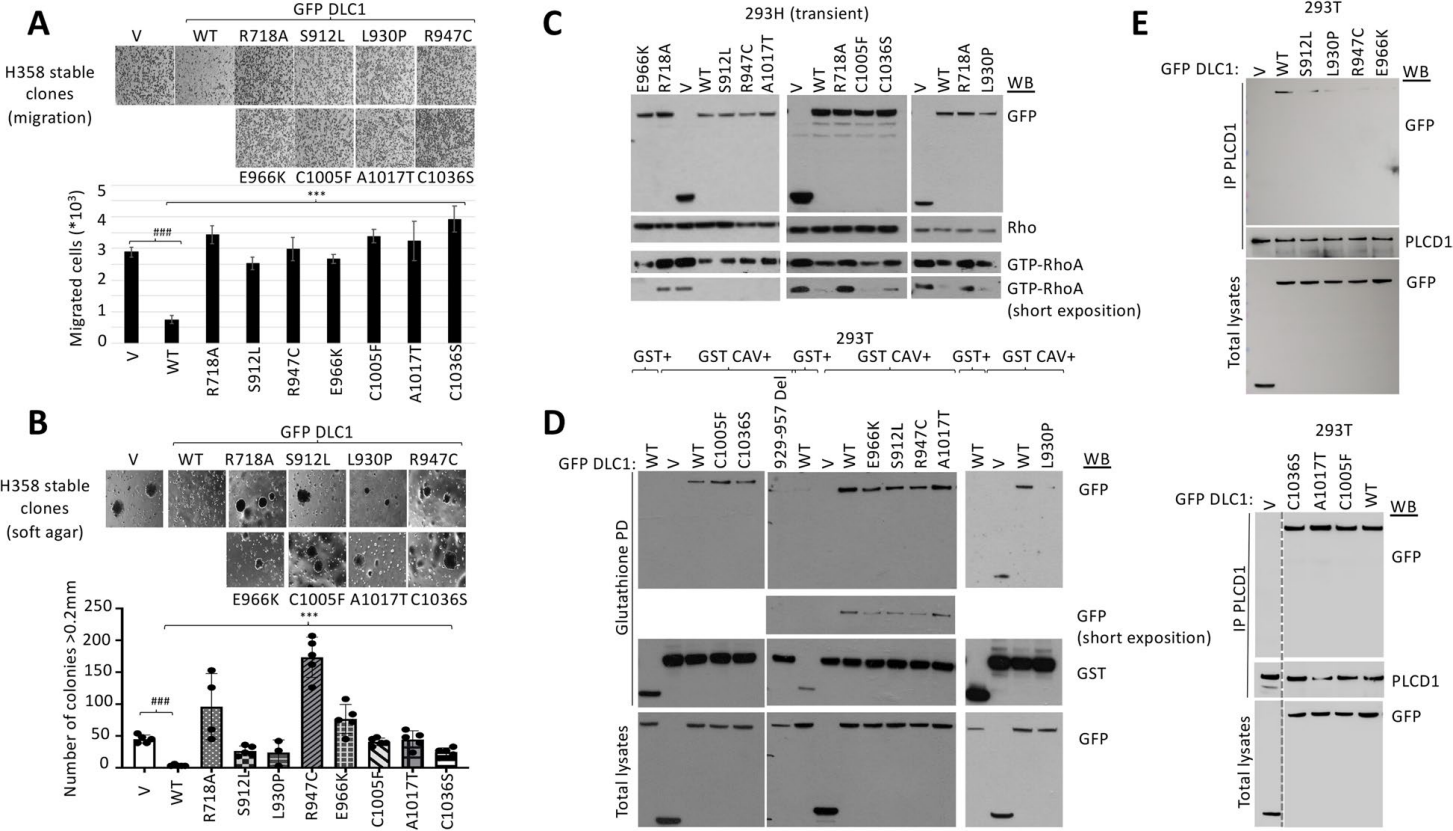
All colorectal-cancer TCGA DLC1 START mutations show impaired inhibition of migration and anchorage-independent growth, and deficient binding to some or all START binding partners, but intact RhoGAP activity. **A** Migration in transwells of H358 control (V) or DLC1 wild type (WT) and mutants stable clones. Migrated cells were fixed and photographed (upper panel), and the number of migrated cells were quantified using Image J, from a total of four images from each duplicate experiment (bottom panel). Bars represent mean +/−SD. t-test was performed for statistical analysis (***, ^###^:*p* < 0.001) **B**. Same stable clones as in A were seeded in soft agar and allow to form colonies for approximately 28 days, and those colonies > 0.2 mm were photographed (upper panel) and counted (lower panel). Each dot represents one plate of cells. 3–5 plates of each stable clone were used for the experiment. Bars represent mean +/− SD. t-test was performed for statistical analysis (***:*p* < 0.001, ^###^:*p* < 0.005) **C**. Active Rho (GTP-RhoA) in 293H control (V) or DLC1 wild type (WT) and mutants transient transfectants was analyzed by Rhotekin pull down assays, followed by RhoA Western blot (WB).GFP DLC1 and total Rho expression were also checked by WB. **D** Glutathione Pull-down (PD) in 293 T cells overexpressing GST control or GST Caveolin-1 (GST CAV) and GFP control (V) or GFP DLC1 wild type (WT) and mutants followed by Western blot (WB) using GFP and GST antibodies. Correct expression of GFP fusion proteins was checked in total lysates (bottom panel). **E** PLCD1 immunoprecipitation (IP) in 293 T cells overexpressing GFP control (V) or GFP DLC1 wild type (WT) or mutants followed by western blot (WB) using the indicated antibodies. The expression of GFP fusion proteins was checked by Western blot in total lysates (bottom panels)

An analysis of the TCGA database for *DLC1* mutants revealed that missense mutations occur frequently and are distributed along its entire coding region, including the START domain [3]. We selected 7 missense mutations from TCGA colorectal cancer (Table 1) and created isogenic stable clones of the different GFP-tagged mutant DLC1 proteins in H358 cells (Supplementary Figure 5). The clones were analyzed for two biological functions - inhibition of migration and colony formation in soft agar, using DLC1 wild type as the positive control and a “GAP-dead” mutant (DLC1 R718A) as the negative control. All mutants were found to be less efficient than wild type in reducing cell migration (Fig. 5A) and anchorage-independent cell growth (Fig. 5B), although the degree of reduction was heterogeneous. In contrast to these impaired biological activities, each of the DLC1-START mutants, when transiently transfected in 293H cells, was found to reduce Rho-GTP levels, as determined by Rhotekin pull-down assay, almost as efficiently as DLC1 WT, unlike the DLC1 “GAP-dead” mutant (DLC1 R718A) (Fig. 5C). Taken together, these results indicate that mutations in the START domain affect DLC1 functions in a RhoGAP-independent manner.

**Table 1:** Summary of the colon cancer-associated DLC1-START domain mutations. TCGA colon cancer associated DLC1 START domain mutations and biological properties analyzed in this study

DLC1 mutation	Biological inhibition relative to DLC1 WT	RhoGAP activity relative to DLC1 WT	Caveolin-1 binding	PLCD1 binding	PS binding	Sensitivity to PS changes
**S912L**	reduced	similar	deficient	deficient	normal	normal
**L930P**	reduced	similar	deficient	deficient	normal	normal
**R947C**	reduced	similar	deficient	deficient	deficient	deficient
**E966K**	reduced	similar	deficient	deficient	deficient	not assayed
**C1005F**	reduced	similar	normal	normal	deficient	not assayed
**A1017T**	reduced	similar	normal	normal	deficient	not assayed
**C1036S**	reduced	similar	normal	normal	deficient	deficient

### DLC1-START mutants have reduced binding to PS, Caveolin-1, and/or PLCD1

As previous research had determined that a DLC1 mutant with a 29 amino acid deletion (amino acids 929–957) in the START domain had decreased Caveolin-1 binding, decreased biological activity, and normal RhoGAP activity [13], we examined the ability of the colorectal cancer-associated DLC1-START mutants to bind Caveolin-1. To that end, we co-transfected 293 T cells with GFP-DLC1 WT or the START domain mutants together with a plasmid encoding a GST-tagged version of Caveolin-1. Two negative controls for the interaction were DLC1 deletion mutant 929–957 and a previously characterized DLC1 cancer-associated point mutant E996K, whose mutation lies just downstream from the amino acids in the deletion mutant. Both mutants had a similar phenotype, including decreased Caveolin-1 binding and tumor suppressor function but efficient RhoGAP activity [3]. The GST pull-downs indicated that the three START domain mutants, S912L, L930P, and R947C, whose lesions lie within or in close proximity to the missing amino acid region in the 929–957 deletion mutant displayed impaired binding to Caveolin-1 compared to DLC1 WT (Fig. 5D, Table 1). However, the three START domain mutants whose lesions are C-terminal to E966K, namely C1005F, A1017T, and C1036S bound Caveolin-1 as efficiently as DLC1 WT (Fig. 5D, Table 1).

As the wild type START domain binds either Caveolin-1 or PLCD1, we evaluated PLCD1 binding in 293 T cells of all DLC1-START mutants (Fig. 5E). PLCD1 immunoprecipitations in cells overexpressing the GFP-tagged DLC1 mutant or WT proteins indicated that the mutants with reduced DLC1-Caveolin-1 binding also had reduced DLC1-PLCD1 binding, while the mutants with wild type binding to Caveolin-1 did not have impaired PLCD1 binding. These results implied there were at least two groups of cancer-associated START mutants: one with reduced Caveolin-1 and PLCD1 binding (S912L, L930P, R947C, E966K) and the other in which Caveolin-1 and PLCD1 binding was intact (C1005F, A1017T, and C1036S).

**Fig. 6:**
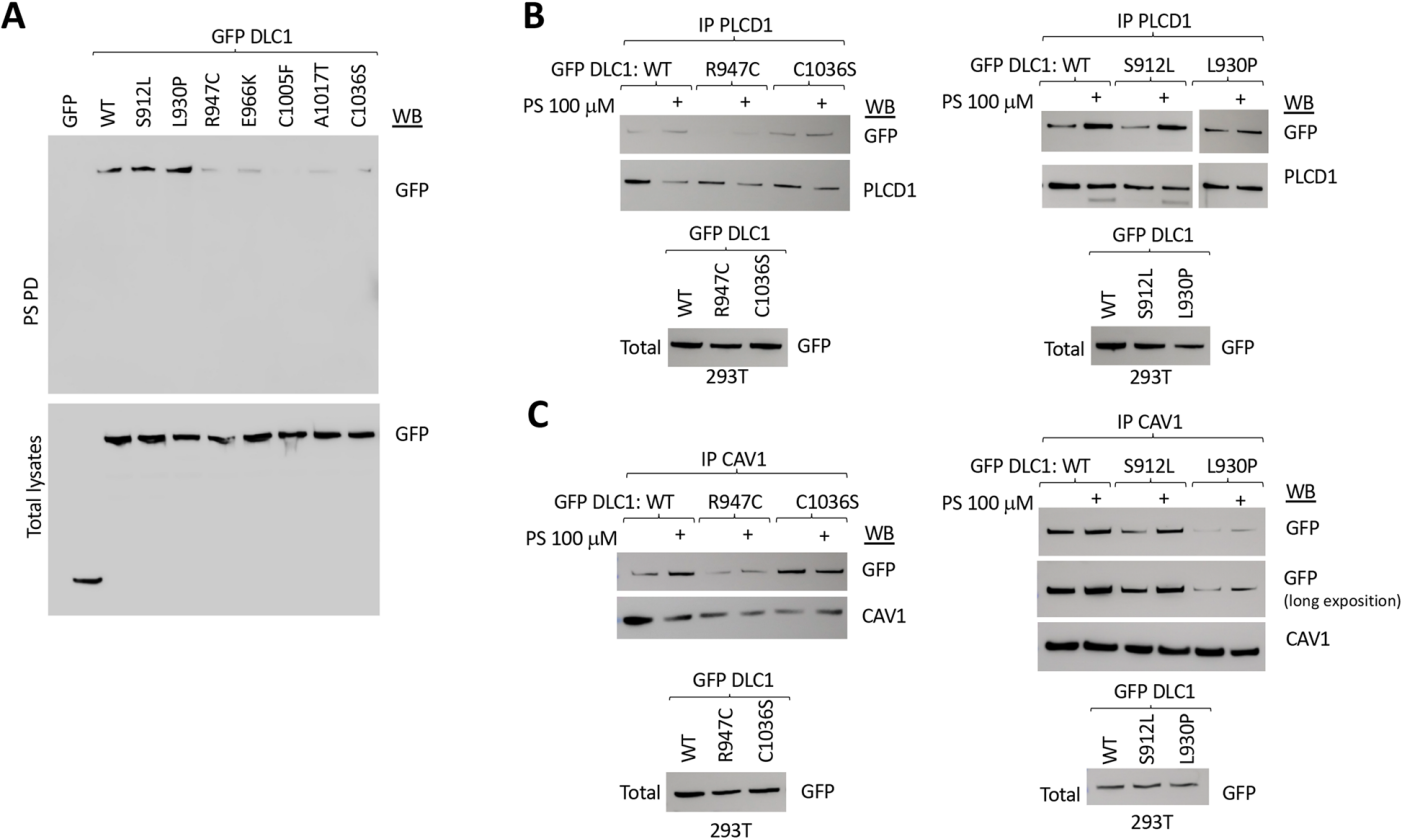
Most DLC1 START mutants show reduced binding to PS and PS supplementation does not improve their binding to Caveolin-1 or PLCD1. **A** Pull-down (PD) using PS-coated beads in 293 T cells overexpressing GFP or GFP DLC1 wild type (WT) or mutants followed by GFP western blot (WB). Expression of GFP fusion proteins was checked by western blot in total lysates (bottom panel). **B**, **C** Immunoprecipitation (IP) using PLCD1 antibody (top) or Caveolin-1 antibody (bottom) in 293 T cells overexpressing GFP DLC1 wild type (WT) or mutants in the presence or absence of 100 μM PS added to the binding reaction, followed by Western blot (WB) using the indicated antibodies. The expression of GFP fusion proteins was checked by Western blot in total lysates (bottom panel)

We also analyzed the START mutants for their ability to bind PS by PS pull-down assays (Fig. 6A). Two of the mutants, S912L and L930P, whose interaction with Caveolin-1 and PLCD1 was reduced, showed efficient binding to PS. In contrast to these two mutants, the other 5 mutants, whose lesions lie C-terminal to L930, displayed impaired binding of DLC1 to PS-coated beads (Fig. 6A). These 5 mutants can be divided into two groups: R947C and E966K, which are deficient for Caveolin-1, PLCD1, and PS binding; and C1005F, A1017T, and C1036S, which are deficient for PS binding but have normal Caveolin-1 and PLCD1 binding (Table 1).

We also evaluated the binding of the isolated START domain to PS in two of the mutants: one mutant in the N-terminal region of DLC1-START (R947C), which showed reduced DLC1 binding to Caveolin-1 and PLCD1, and one in the C-terminal region of DLC1-START (C1036S), which had wild-type binding to Caveolin-1 and PLCD1. PS pull-down assays (Supplementary Figure 6A) showed both mutants bound PS-coated beads less efficiently than WT, reproducing the results from DLC1 full length mutants. Similar results were also seen for the two mutants by lipid overlay assays in two different cell systems (mammalian 293 T cells and insect S2 cells), using different concentrations of immobilized PS and immunoprecipitated GFP-DLC1 START domain WT or mutants (Supplementary Figure 6B).

To examine whether some of the START mutants might have lost their ability to respond to changes in PS concentration, we evaluated the interaction between GFP-tagged DLC1 and endogenous Caveolin-1 or PLCD1 by immunoprecipitation assays performed with PS (100 μM) being added to the binding reaction (Fig. 6B,C). When two of the mutants that were deficient for direct PS binding were examined (R947C and C1036S), the addition of PS did not substantially increase the binding between the full-length DLC1 and PLCD1 (Fig. 6B) or Caveolin-1 (Fig. 6C), nor did it modify their interaction with the isolated mutant START domains (Supplementary Figure 6C), in contrast to the wild type. On the other hand, mutants with efficient PS binding (S912L and L930P) were able to respond to the exogenous addition of PS by increasing their binding to both PLCD1 (Fig. 6B) and Caveolin-1 (Fig. 6C).

### Mapping cancer-associated mutations in a structural model of DLC1-START

**Fig. 7:**
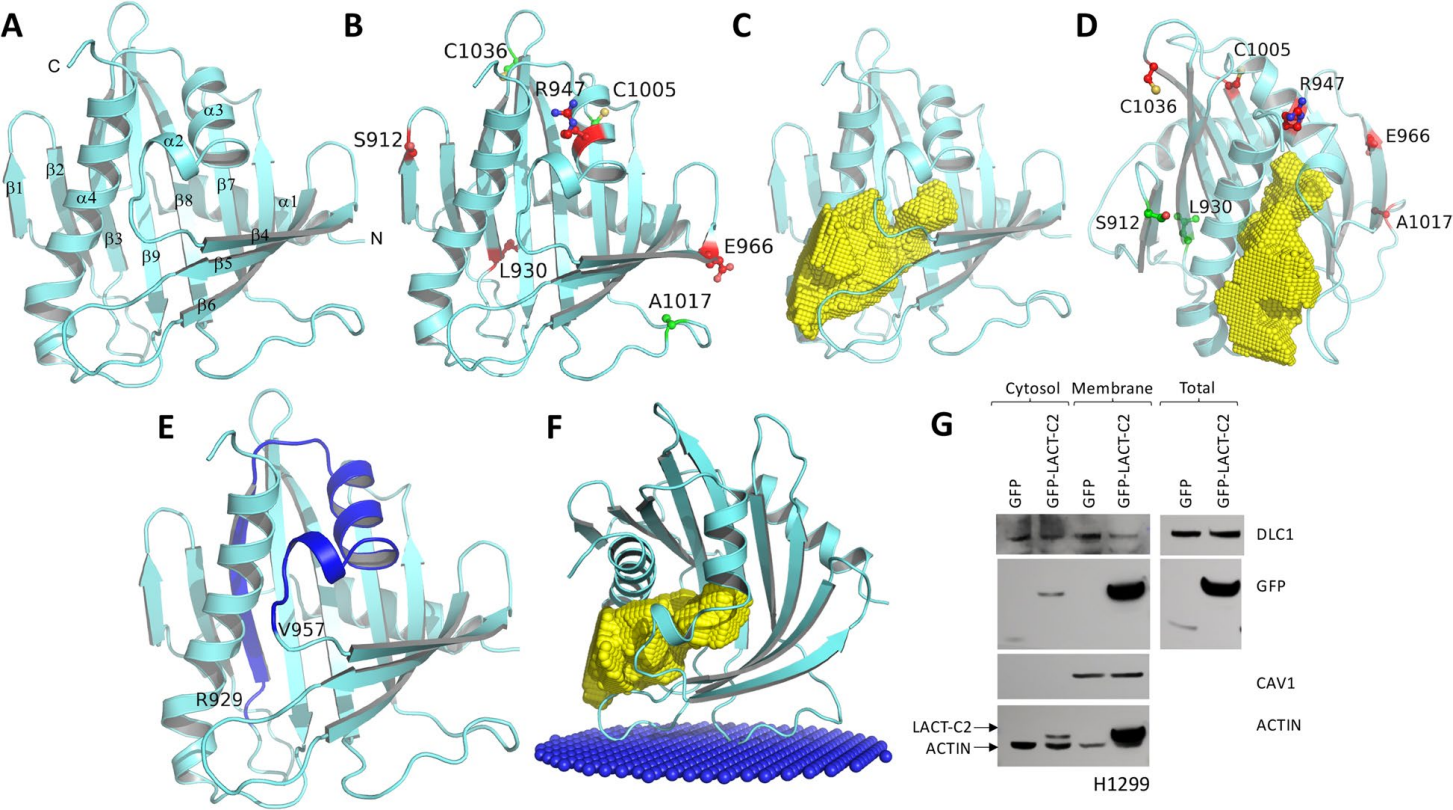
Mapping cancer-associated mutations in the homology model of the DLC1-START domain and structural analysis suggesting the putative binding site of lipid (PS) and interacting proteins. **A** Cartoon representation of the homology model of the DLC1-START domain, generated using the DLC2-START domain as a template. N- and C-terminal ends are indicated. **B** Cancer-associated mutations mapped on the DLC1-START domain that has normal (green) and impaired (red) binding to Caveolin-1 and PLCD1. **C** Analysis of cavity and tunnels in the model of the DLC1-START domain shows the potential binding site of the lipid (PS). The empty space that forms this tunnel is shown in yellow. **D** Cancer-associated mutations mapped on the DLC1-START domain that show normal (green) and impaired (red) binding to PS. **E** Model of the DLC1-START domain with residues from 929 to 957 highlighted in blue. The deletion of this region has been shown to abolish the DLC1-Caveolin-1 interaction. **F** Model showing orientation and positioning of the DLC1-START domain at the membrane. The computational approach was used to identify residues involved in the initial docking of the DLC1-START domain with the membrane interface. The lipid headgroups of the membrane bilayer that point towards the cytoplasm are shown as blue colored spheres**. G** Western blot in cytosolic, membrane and total cell lysates from H1299 cells transfected with GFP or the C2 domain of Lactadherin, using the indicated antibodies

To better understand the structural implications of the cancer-associated mutations in DLC1-START and the role of these mutations in DLC1 interaction with PS, Caveolin-1, and PLCD1, we built a homology model of DLC1-START, using DLC2-START as a template for homology modeling, as its crystal structure, which has been reported [21], shares 57% sequence identity and 73% sequence similarity with DLC1-START (Supplementary Figure 7A). As with DLC2-START, the overall structure of DLC1-START contains a nine-stranded (β1-β9) curved antiparallel β-sheet packed tightly around a C-terminal α-helix (α4), consistent with a helix-grip type fold (Fig. 7A and Supplementary Figure 7B).

The seven cancer-associated mutations (Table 1) mapped on the structure of DLC1-START are shown in Fig. 7B. They are located on different secondary structures in DLC1-START (Supplementary Figure 8). R947 is located on α2, and its mutation is likely to have a significant effect on the tertiary structure. A1017 is present in the loop between β7-β8 strands, while the five remaining mutations are located either at the beginning or end of different β-strands present in the central β-sheet; their mutations are likely to perturb local interactions around these mutant residues.

### Structural insights into the role of DLC1-START mutations in its interaction with PS, Caveolin-1, and PLCD1

The cavity analysis in the model of DLC1-START showed a large lipid-binding pocket in the concave side of the curved β-sheet, as seen in other START domain structures (Fig. 7C). The presence of such a large cavity suggests that DLC1 probably uses this site to bind to its partner lipid. Structural comparison of DLC1-START/DLC2-START with the START domains of ceramide-transfer protein (CERT) and phosphatidylcholine transfer protein (PCTP) shows a lack of conservation of amino acids that contribute to lipid headgroup specificity. The presence of multiple polar and charged side chain-containing amino acids in the cavity of DLC1-START near the lipid head-group binding site further supports the charged PS lipid as a natural ligand for this protein. Two mutations located near the N-terminus (S912L and L930P) show normal binding to PS, whereas the other five mutations, located in the central β-sheet around the PS binding pocket, are deficient in PS binding (Fig. 7D). A recent bioinformatic report that included the DLC1 START 3D modelled structure positioned R947 close to two other residues (S1077, F1078), presenting the possibility that they may interact and function together at the tertiary level with the R947C mutation, significantly destabilizing the START domain [30].

The DLC1-START deletion mutant (residue 929–957) has been shown to result in decreased Caveolin-1 binding [13]. Residues 929–957 form β3-strand and α2 and α3 helices in DLC1-START, so deletion of these residues is likely to destabilize the helix-grip fold (Fig. 7E). Mutations in residues L930 and R947 present in this segment are also deficient in binding to Caveolin-1/PLCD1 (Fig. 7B). The other two DLC1 mutants (S912, E966) deficient in binding to Caveolin-1/PLCD1 are not limited to one part of the protein, suggesting that these mutations may affect the DLC1/Caveolin-1/PLCD1 interaction indirectly, either by affecting PS binding or possibly by destabilizing the helix-grip fold.

### PS may be important for DLC1 recruitment to the membrane

To gain insight into the orientation and position of DLC1-START at the membrane, we carried out modeling analysis [24], which showed that the loops present between β-strands β4-β5 and β6-β7 are involved in the DLC1-membrane interaction (Fig. 7F). In this model, the cavity where PS is expected to bind is located close to the DLC1-membrane interface.

Given this observation as well as the effect of PS on DLC1 functionality and on its interaction with Caveolin-1 or PLCD1, we asked whether PS may induce the recruitment of DLC1 to the membrane, where it would therefore bind more efficiently to Caveolin-1 or PLCD1. For that purpose, we performed cytosol and membrane fractionation studies in H1299 cells, which express considerable amounts of endogenous DLC1, in the presence of the C2 domain of Lactadherin (Fig. 7G). The sequestration of PS in the membrane by Lactadherin specifically reduced the pool of DLC1 in the membrane, but not in the cytosolic compartment. These results suggest PS contributes to DLC1 membrane localization.

## Discussion

The current report has made several noteworthy observations about the DLC1 START domain. First, we provide evidence of two previously unreported interactions with it, one with a lipid, phosphatidylserine (PS), and the other with a protein, PLCD1. These findings expand the versatility of START domains, as DLC1-START is the first found to bind PS, which promotes the binding to other proteins. Second, we determined the TCGA colorectal cancer database contains several cancer-associated *DLC1* point mutants whose mutations lie in the START domain and analyzed 7 of them. Each mutant is deficient for the biological activities of cell migration and anchorage-independent growth while retaining its ability to reduce intracellular Rho-GTP levels. Third, our analyses have enabled us to conclude that PS binding to the START domain enhances the interaction between the START domain and its two protein ligands, and that all 7 DLC1-START mutants are deficient for binding to at least one of its interacting partners. Finally, analysis of a structural model of DLC1-START has provided insight into these macromolecular interactions and how they may be perturbed by the mutants.

Our results demonstrated that DLC1 cooperates with PLCD1 to inhibit migration, and the colorectal cancer-associated DLC1 START mutants that displayed reduced binding to PLCD1 had impaired DLC1 tumor suppressor functions, suggesting the interaction with PLCD1 is essential for the full tumor suppressor function of DLC1. Binding experiments identified two PLCD1-interacting regions in the DLC1 protein: one in the N-terminal region (amino acids 80–400) and a region in the C-terminal end (amino acids 848–1091) that is mainly composed of the START domain (amino acids 878–1081). Our interaction studies in cells overexpressing the DLC1-START, as well as cell migration and colony formation in soft agar experiments with the DLC1-START mutants, indicated that an intact START domain is sufficient and essential for PLCD1 binding. However, we have not ruled out a possible additional contribution of the binding of PLCD1 to the DLC1 N-terminal fraction to the overall effect of DLC1 activity by PLCD1.

We identified two types of interaction between DLC1, PLCD1, and Caveolin-1. In one type, Caveolin-1 is able to bind PLCD1 independently of DLC1 in cells. In a second type, DLC1-START can bind both PLCD1 and Caveolin-1. However, our competition studies indicated that a single START domain binds only PLCD1 or Caveolin-1 at the same time, rather than binding both proteins simultaneously. The latter biochemical results were correlated with the observation that while PLCD1 or Caveolin-1 alone cooperated with DLC1 in the inhibition of cell migration, co-expression of PLCD1 and Caveolin-1 together in cells did not result in further inhibition.

The 15 genes that encode START domains have been grouped into 6 subfamilies, one of which is composed of DLC1, DLC2, and DLC3 [31]. The analyses of the limited number of START domains whose lipid ligand has been identified indicate that each START domain may bind one or more closely related lipids and that START domains from different subfamilies can clearly bind distinct lipids. Our preliminary lipid overlay assays, using GFP-DLC1–848-1091 as a bait, identified three possible DLC1-START interacting lipids: phosphatidylcholine (PC), cardiolipin, and phosphatidylserine (PS). The localization of PC and cardiolipin to the outer layer of the lipid membrane or to the mitochondrial membrane [32], respectively, made these two candidates very unlikely to bind DLC1 in a physiologic scenario.

Additional experiments demonstrated that the DLC1 START domain binds PS in cells, and this binding promotes the binding of DLC1 to PLCD1 or to Caveolin-1. Although Caveolin-1 and the C2 domain of PLCD1, which was identified in this work as one of the interacting regions with DLC1-START, are known to bind PS [25, 27], our data indicated that DLC1 can bind PS regardless of the presence of Caveolin-1 and PLCD1 in the cell, as shown by in vitro PS binding assays and PS pull-down experiments in cells that do not express either Caveolin-1 or PLCD1. Thus, PS is a highly plausible physiologic interacting lipid for DLC1-START. Moreover, we also detected the binding of DLC2 and DLC3 to PS, suggesting that PS binding is a general property of the DLC protein family.

Experiments designed to manipulate the amount of PS available in cells revealed that the binding of DLC1 to PS is a critical element for DLC1-mediated inhibition of migration and proliferation. The alteration of PS levels in the cell has been linked to changes in the subcellular targeting of proteins to the membrane [18]. One interesting example is that of Rac1. Rac1 GTPase activity is independent of PS [33], but the stimulation of cells with PS induces the association of Rac1 to the plasma membrane, consequently affecting cytoskeletal rearrangement and cell migration [34]. Similarly, our data indicate that some cancer-associated DLC1 START mutants bind PS less efficiently than DLC1 WT, and that they display impaired inhibition of migration and anchorage-independent growth, without affecting DLC1 RhoGAP activity. Moreover, we demonstrated that the stimulation of cells with PS induces the association of DLC1 with Caveolin-1 and/or PLCD1. To our knowledge, this is the first START domain shown to bind a protein, Caveolin-1 or PLCD1, and for which the lipid binding promotes the interaction with the protein. Our preliminary studies with DLC2 and DLC3 suggest that these characteristics may be shared by the START domains of all three proteins.

As in other START containing proteins, we believe that the PS binds to DLC1 in the hydrophobic tunnel of that domain. At this time, we have not resolved if it is also involved in abstraction and movement of PS. The nature of the small hydrophobic pocket in DLC1 and other START domains makes it unlikely to bind more than one lipid molecule at a time. In addition, the lipid binding cavity may not be wide enough to accommodate a portion of PLCD1 or Caveolin-1 to bind other residues of the PS that is bound to DLC1-START. Therefore, it is possible that PS recognition by DLC1 exerts a regulatory function by promoting its subcellular localization to the plasma membrane, where it interacts with other effectors such as Caveolin-1 or PLCD1, without affecting DLC1 RhoGAP activity, in a manner analogous to the model for the effect of PS on Rac1 activity [34]. In agreement with that hypothesis, our preliminary fractionation studies showed that the sequestration of PS in the cell by the presence of the C2 domain of Lactadherin reduced the amount of DLC1 specifically in the membrane. The proposed DLC1-START model presented here predicts the PS-interacting region to be located close to the DLC1-membrane interface, but co-crystallization studies and the recognition of key interacting residues in DLC1-START domain for PS, Caveolin-1, and PLCD1 could increase understanding the stoichiometry of these interactions.

We identified three classes of DLC1 START domain mutants among the seven TCGA colorectal cancer mutants analyzed (Table 1). One class (S912L, L930P) is deficient for Caveolin-1 and PLCD1 binding, but binds PS efficiently. A second class (R947C, E966K) shows decreased binding to Caveolin-1, PLCD1, and PS. The third class (C1005F, A1017T, and C1036S) retains intact binding to Caveolin-1 and PLCD1, while its binding to PS is reduced. According to our structural model of DLC1-START, most of our PS-deficient mutants were present in the central β-sheet around the PS binding pocket, therefore explaining their impaired binding. On the other hand, a previous study proposed that the C-terminal helix START domains would undergo a folding/unfolding transition during lipid binding. This transition is thought to gate the lipid binding site, and the importance of this mechanism was supported by the effects on lipid binding of some of the mutations in STARD1 appearing in this region [35]. The location of R947C in the predicted model of DLC1-START in this area may account for the reduced lipid binding ability observed for this mutant.

**Fig. 8:**
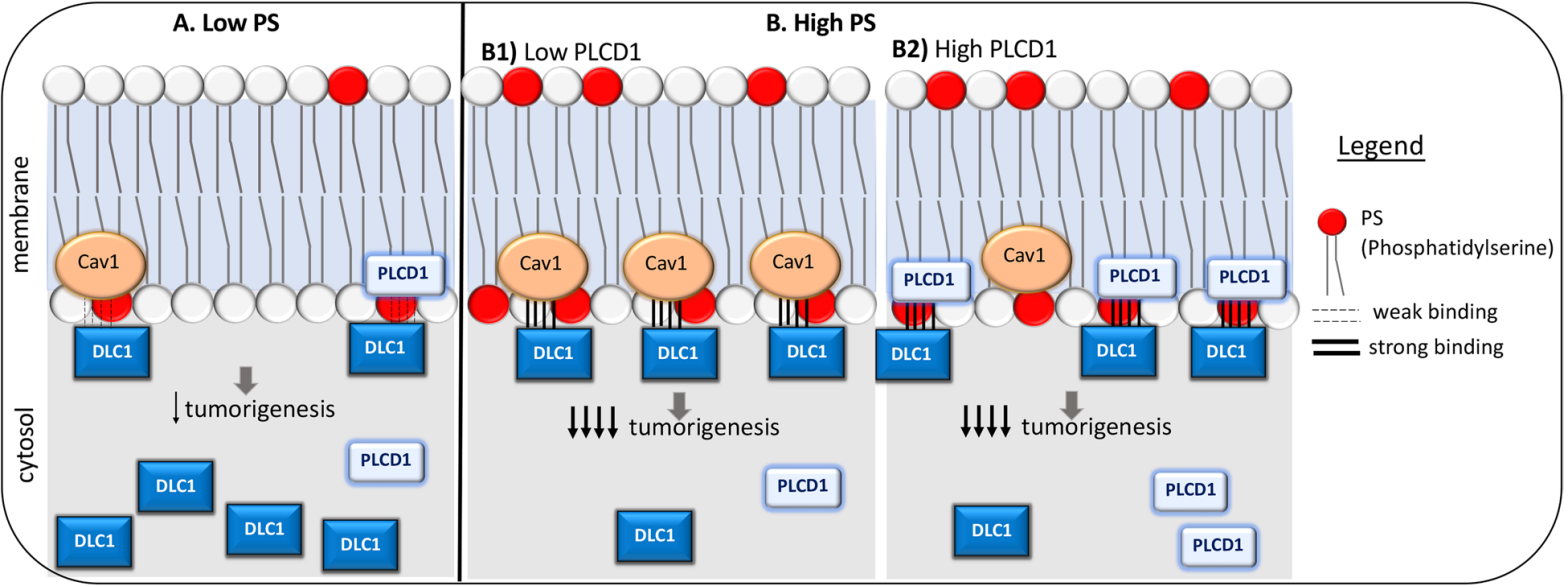
Simplified graphical summary of working hypothesis. **A** Under low phosphatidylserine (PS) conditions, most DLC1 is present in the cytosolic compartment. The pool of DLC1 in the membrane (if any) may bind the existing Caveolin-1 (Cav1), or Phospholipase C delta 1 (PLCD1), although we hypothesize that this binding is weak. **B** High PS levels promotes the recruitment of DLC1 to the membrane. **B1)**. At high PS levels, Cav1 presence in the membrane is higher, and the membrane-recruited DLC1 interacts more efficiently to the existing Cav1. Therefore, DLC1 inhibition of tumorigenesis (migration, anchorage-independent growth) is stronger. **B2).** When PLCD1 levels are increased, more PLCD1 is recruited to the membrane. Competition between PLCD1 and Cav1 for DLC1 binding exists, displacing the DLC1/Cav1 interaction. DLC1 inhibition of tumorigenesis is still strong. *Not represented: At low PS, there is still competition between Cav1 or PLCD1 to bind DLC1. At high PS levels and high PLCD1 levels, Cav1 still binds PS, but at other regions of the membrane where PLCD1 is not binding*

In summary, we have identified PS as a feasible physio-logical DLC1-START interacting lipid that is critical for DLC1 full activity, possibly by anchoring DLC1 to the membrane and promoting its binding to other proteins, such as Caveolin-1 or PLCD1. Overall, the interaction between DLC1-START, Caveolin-1, PLCD1, and phosphatidyserine (PS) contributes to DLC1 tumor suppressor function(s) in a RhoGAP-independent manner (Fig. 8).

## Conclusions

This report has identified two novel interactions of the DLC1-START domain, one with a lipid (PS), and the other with a protein (PLCD1), that are important for DLC1 functionality. To the best of our knowledge, this is the first START domain shown to bind a lipid that promotes the binding to other proteins. In addition, PS binding contributes to the interaction of DLC1-START with Caveolin-1 and PLCD1, and to the tumor suppressor functions of DLC1 that are RhoGAP-independent. The analysis of several cancer-associated DLC1-START mutants provides strong evidence for the importance of this domain for DLC1 in tumorigenesis. All of the mutants analyzed are deficient for all or some of the interactions we have identified and possess reduced tumor suppressor function, but intact RhoGAP activity. This study highlights the importance of the START domain in DLC1 functions and adds to the understan-ding of its RhoGAP-independent-scaffolding activities.

## Supplementary Information

**Additional file 1.**

## Data Availability

All data that support the findings of this study are available from the corresponding authors upon reasonable request.

## Abbreviations

DLC1, 2, 3: Deleted in liver cancer 1, 2, 3
RhoGAP: Rho GTPase activating protein
START: StAR-related lipid transfer
PLCD1: Phospholipase C delta 1
PS: Phosphatidylserine
PC: Phosphatidylcholine
TCGA: The Cancer Genome Atlas database
WT: Wild type
CPTAC: Clinical Proteomic Tumor Analysis Consortium
