# Supplementary material for: The tumor suppressor activity of DLC1 requires the interaction of its START domain with Phosphatidylserine, PLCD1, and Caveolin-1
Source: Mol Cancer. 2021 Nov 2;20:141. doi: 10.1186/s12943-021-01439-y (PMC8561924; doi:10.1186/s12943-021-01439-y)
Supplement: Supplementary file 1 — Additional file 1. [file 12943_2021_1439_MOESM1_ESM.zip › Sanchez-Solana et al_Supplementary information.docx]

**Supplementary Figure Legends**

**Supplementary Figure 1. DLC1, Caveolin-1 and PLCD1 protein expression are positively correlated in lung cancer.** CPTAC Protein expression levels were analyzed in lung adenocarcinoma (A) and lung squamous cell carcinoma (B) datasets and paired to normal lung (top panel). Expression values for DLC1, Caveolin-1 and PLCD1 in A and B are positively correlated (bottom panel). No: normal. Tu: tumor. The numbers indicate the number of patients in each group.

**Supplementary Figure 2. DLC1-START, DLC2 and DLC3 bind Phosphatidylserine. A.** Lipid overlay binding assay in 293T cells overexpressing GFP or GFP DLC1 START 848-1091 using Echelon commercial lipid binding strips for different membrane lipids. The arrangement of the different lipids is shown on the right. **B.** Lipid overlay binding assay in 293T cells overexpressing GFP or GFP DLC1 START 848-1091 or S2 cells overexpressing a GFP fusion control protein or GFP DLC1 825-1091 using different concentrations of PS spotted onto nitrocellulose membranes. The expression of all GFP fusion proteins was checked by Western blot (left panel). **C.** Lipid overlay binding assay in 293T cells overexpressing GFP, GFP DLC2 or GFP DLC3 using different concentrations of PS spotted onto Hybond-D extra nitrocellulose membranes. The expression of GFP fusion proteins was checked by western blot (left panel).

**Supplementary Figure 3. DLC1 binds PS regardless of Caveolin-1 or PLCD1 expression. A.** Western blot analysis of DLC1, PLCD1, and Caveolin-1 (CAV1) protein expression in some gastric cancer cell lines. H358 cell line was used as a positive control for PLCD1 and Caveolin-1 expression. Actin expression was used as loading control. **B.** Pull-down (PD) using control (CTRL) or PS-coated beads in CRL-1739 cells overexpressing GST, GST Caveolin-1 (CAV1), GST PLCD1 or GST Caveolin-1 + GST PLCD1, followed by DLC1 Western blot (WB). The expression of DLC1 and GST fusion proteins was checked by Western blot in total lysates (bottom panel). **C.** Pull-down (PD) using MBP control or MBP DLC1 START 848-1091 purified proteins in CRL-1739 cells overexpressing GST, GST Caveolin-1 (CAV1) or the C2 domain of PLCD1 (GST PLCD1-C2) in the presence or absence of 100 μM PS, followed by GST western blot. The amount of purified MBP proteins is shown as stained with Ponceau S (bottom panel).

**Supplementary Figure 4. The presence of the C2 domain of Lactadherin reduces the interaction between DLC1 or DLC1-START to Caveolin-1 and PLCD1. A.** Immunoprecipitation (IP) using Caveolin-1 (CAV1) antibody (left panel) or PLCD1 antibody (right panel) in 293T cells overexpressing GFP DLC1 START 848-1091 and GFP, the C2 of Lactadherin (LACT-C2 WT), or a triple mutant (W26A, W33A, F34A) of the C2 form of Lactadherin unable to bind PS (LACT-C2 3A mut), followed by western blot (WB) using the indicated antibodies. The expression of GFP-fusion proteins was checked by Western blot in total lysates (bottom panel). **B.** Immunoprecipitation (IP) using Caveolin-1 (CAV1) antibody (left panel) or PLCD1 antibody (right panel) in 293T cells overexpressing GFP DLC1 and GFP or the C2 domain of Lactadherin (LACT-C2), followed by western blot (WB) using the indicated antibodies. The expression of GFP-fusion proteins was checked by Western blot in total lysates (bottom panel).

**Supplementary Figure 5. Expression of DLC1 in H358 stable clones.** Western blot (WB) analysis of DLC1 protein expression in H358 stable clones overexpressing control GFP (V), GFP DLC1 wild type (WT) or GFP DLC1 mutants. Actin expression was used as loading control.

**Supplementary Figure 6. DLC1 START mutants show reduced binding to PS and PS supplementation does not improve their binding to Caveolin-1 or PLCD1 A.** Pull-down (PD) using control (ctrl) or PS-coated beads in 293T cells overexpressing GFP or GFP DLC1 START 848-1091 wild type (WT) or mutants followed by GFP western blot (WB). The expression of GFP fusion proteins was checked by western blot in total lysates (bottom panel). **B.** Lipid overlay binding assays in 293T (top) or S2 (bottom) cells overexpressing GFP, or GFP DLC1 START 848-1091 (in 293T) or GFP DLC1 825-1091 (in S2) wild-type (WT) or mutants (R947C, C1036S) using different concentrations of PS spotted onto Hybond-C extra nitrocellulose membranes. The expression of GFP fusion proteins was checked by Western blot (WB) (left panel). **C.** Immunoprecipitation (IP) using Caveolin-1 (top) or PLCD1 (bottom) antibodies in 293T cells overexpressing GFP DLC1 START 848-1091 wild type (WT) or mutants (R947C, C1036S) in the presence or absence of 100 μM PS added to the binding reaction, followed by Western blot (WB) using the indicated antibodies. The expression of GFP fusion proteins was checked by Western blot in total lysates (bottom panel).

**Supplementary Figure 7.  Sequence alignment of human DLC1-START and DLC2-START domains, and topology diagram of DLC1-START domain**.

**A**. Structure-based sequence alignment of amino acids present in the START domain of DLC1 and DLC2 proteins. Identical and similar residues are highlighted in black and gray, respectively.

**B**. Topology diagram of DLC1-START domain showing arrangement of secondary structural elements. Arrows represent β-strands, whereas cylinders represent α-helices. The residue numbers at both ends of the secondary structural elements are given. The position of cancer-associated mutations is shown as a yellow star.

**Supplementary Figure 8**. **Intramolecular interactions formed by DLC1-START residues that are mutated in cancer.** Mutated residues are shown in orange color, whereas neighboring residues are colored cyan. The dotted line shown in gray color shows hydrogen bonds and salt-bridge interaction.

**Supplementary Table 1. Primers used for cloning and mutagenesis.**

| **Primer** | **Sequence (5’-3’)** |
| --- | --- |
| **GFP DLC1 848-1091** | GCAGCAGAATTCAGTTCCCGAGGAAATGAGCCG (F) |
|  | TAAGCAGTCGACTGATCACCTAGATTTGGTGTCTTTGG (R) |
| **MBP DLC1 848-1091** | TAAGCAGAATTCATGGTTCCCGAGGAAATGAGCCG (F) |
|  | TAAGCAGTCGACTGATCACCTAGATTTGGTGTCTTTGG (R) |
| **PLCD1 1-756** | TAAGCAGGTACCCAGTGCCTGGGGATCCGGAGC (F) |
|  | TAAGCAGCGGCCGCCTAGTCCTGGAGGGAGATC (R) |
| **PLCD1 1-140** | TAAGCAGGTACCCAGTGCCTGGGGATCCGGAGC (F) |
|  | TAAGCAGCGGCCGCTTACTGGGCATCAGCTGGCG (R) |
| **PLCD1 140-300** | TAAGCAGGTACCATGCACTGGGTGCTGGGGC (F) |
|  | TAAGCAGCGGCCGCTTATAAGTACATGAGGAAGCC (R) |
| **PLCD1 300-450** | TAAGCAGGTACCATGCTGTCGGCTGACGGC (F) |
|  | TAAGCAGCGGCCGCTTAGCTCAGGGGAGGGCAGGC (R) |
| **PLCD1 450-600** | TAAGCAGGTACCATGAACTGAAGGGGAAGATCC (F) |
|  | TAAGCAGCGGCCGCTTAGGTACACGTCCATCTCTGG (R) |
| **PLCD1 600-756** | TAAGCAGGTACCATGAGGGCCGCTTCCAGGACAACG (F) |
|  | TAAGCAGCGGCCGCCTAGTCCTGGAGGGAGATC (R) |
| **DLC1 S912L** | CAGCTACTCCACTTTGGAGCAGGCTGAGC (F) |
|  | GCTCAGCCTGCTCCAAAGTGGAGTAGCTG (R) |
| **DLC1 L930P** | GAAGGACCCCCTCTGAGGCCATGGAGGTCAGTCATTGAAG (F) |
|  | CTTCAATGACTGACCTCCATGGCCTCAGAGGGGGTCCTTC (R) |
| **DLC1 R947C** | GCCAGAGGAAATCTTAAAGTGCCTACTTAAAGAACAGCAC (F) |
|  | GTGCTGTTCTTTAAGTAGGCACTTTAAGATTTCCTCTGGC (R) |
| **DLC1 C1005F** | CTAATTTACCCAAAGGAGCCTTCGCCCTTTTACTAACCTCTGTG (F) |
|  | CACAGAGGTTAGTAAAAGGGCGAAGGCTCCTTTGGGTAAATTAG (R) |
| **DLC1 A1017T** | CTGTGGATCACGATCGCACACCTGTGGTGGGTGTG (F) |
|  | CACACCCACCACAGGTGTGCGATCGTGATCCACAG (R) |
| **DLC1 C1036S** | GGTATTTGATTGAACCCAGTGGGCCAGGAAAATCC (F) |
|  | GGATTTTCCTGGCCCACTGGGTTCAATCAAATACC (R) |
