# Supplementary figures and images for: The tumor suppressor activity of DLC1 requires the interaction of its START domain with Phosphatidylserine, PLCD1, and Caveolin-1
Source: Mol Cancer. 2021 Nov 2;20:141. doi: 10.1186/s12943-021-01439-y (PMC8561924; doi:10.1186/s12943-021-01439-y)

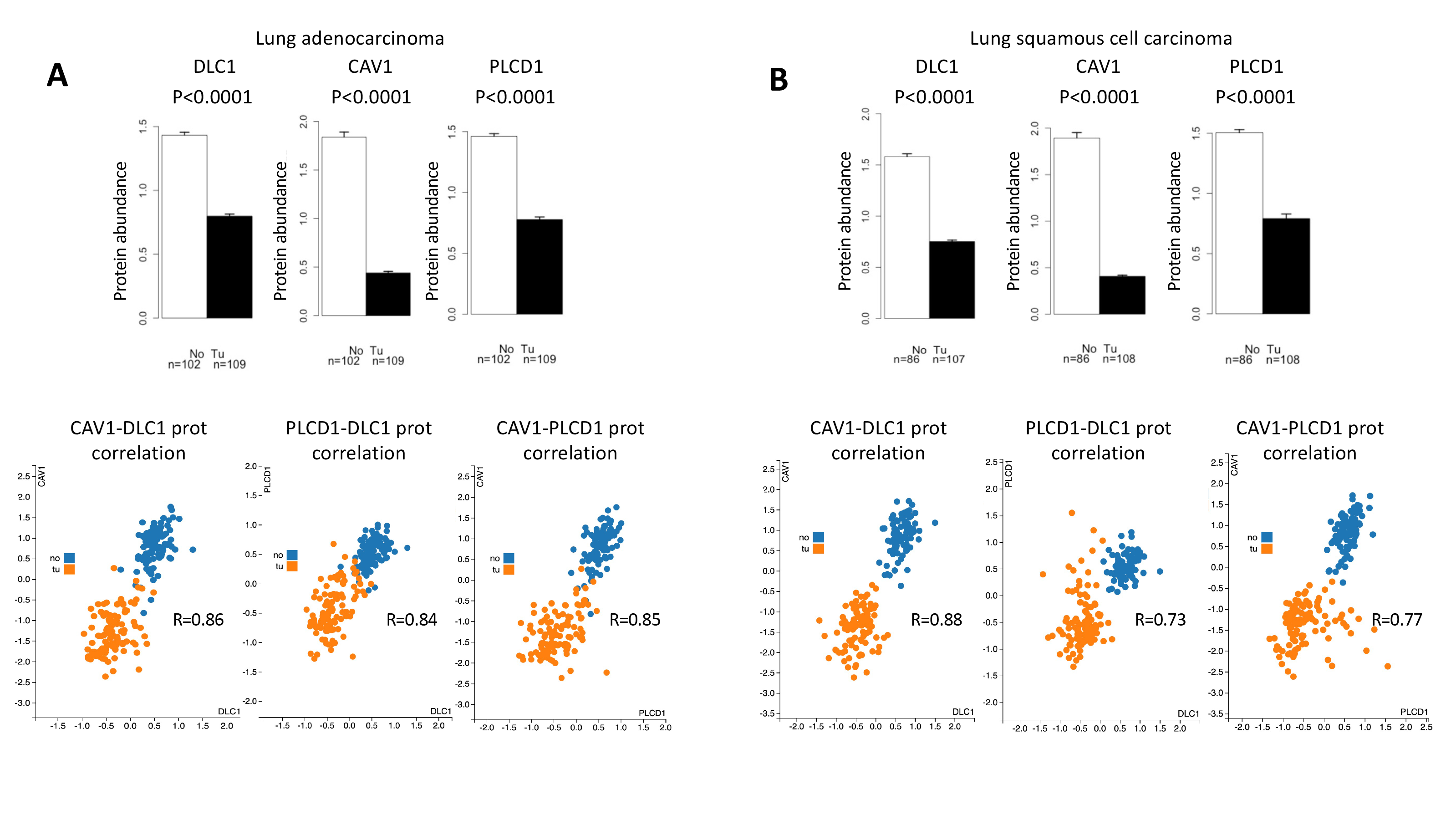

Supplement: Supplementary file 1 — Additional file 1. [file 12943_2021_1439_MOESM1_ESM.zip › Suppl fig 1.png]

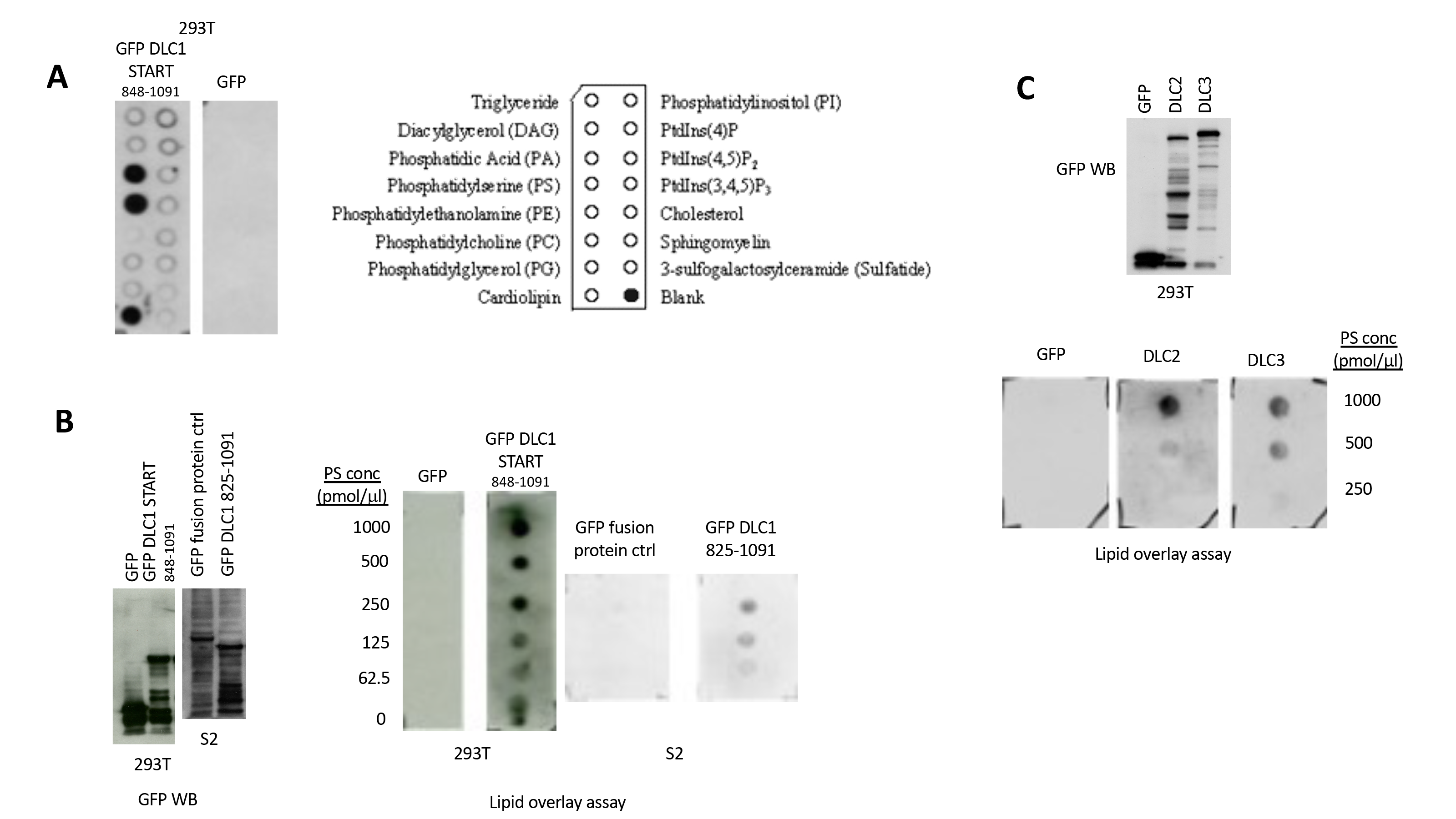

Supplement: Supplementary file 1 — Additional file 1. [file 12943_2021_1439_MOESM1_ESM.zip › Suppl Fig 2.png]

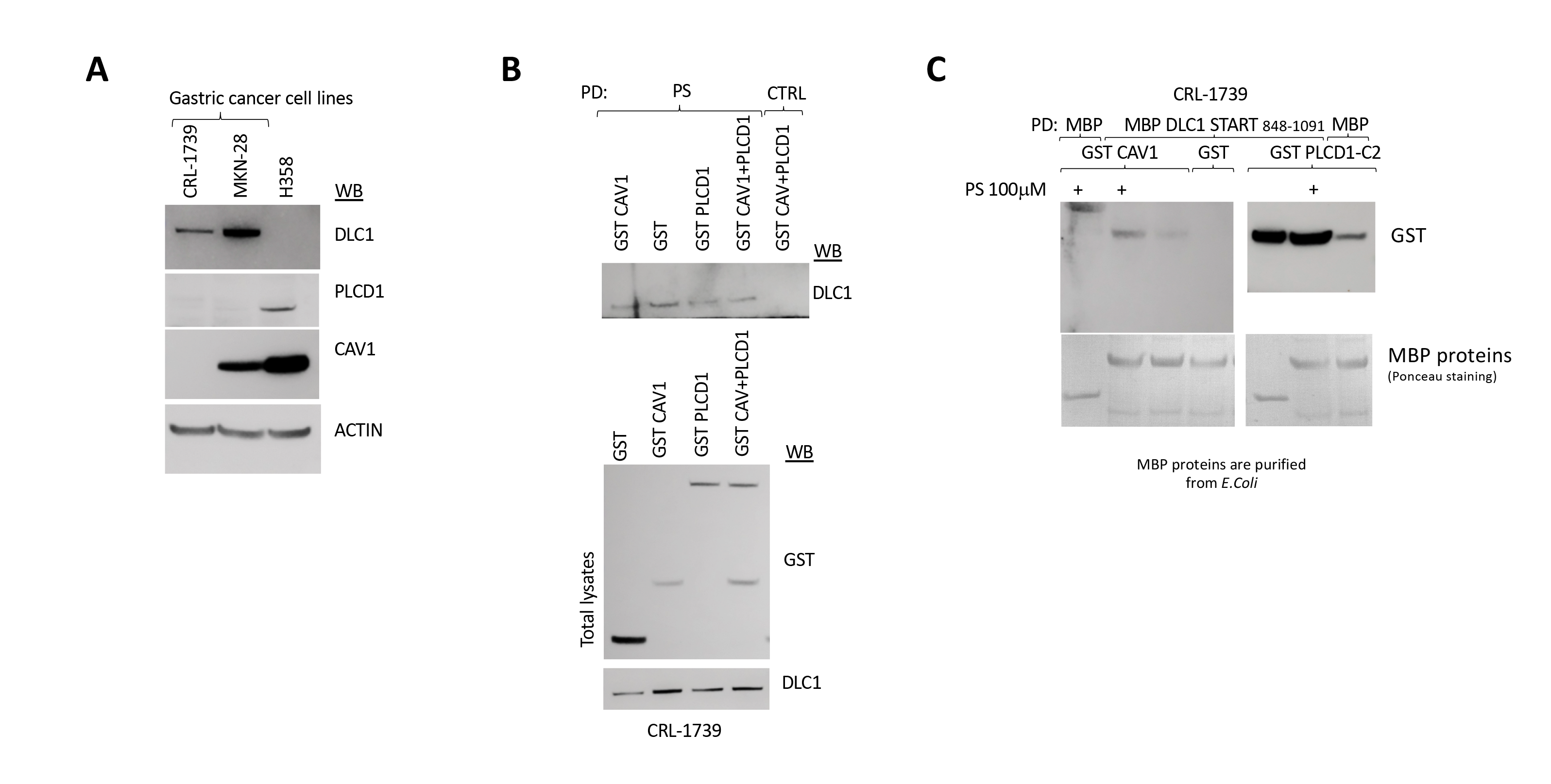

Supplement: Supplementary file 1 — Additional file 1. [file 12943_2021_1439_MOESM1_ESM.zip › Suppl Fig 3.png]

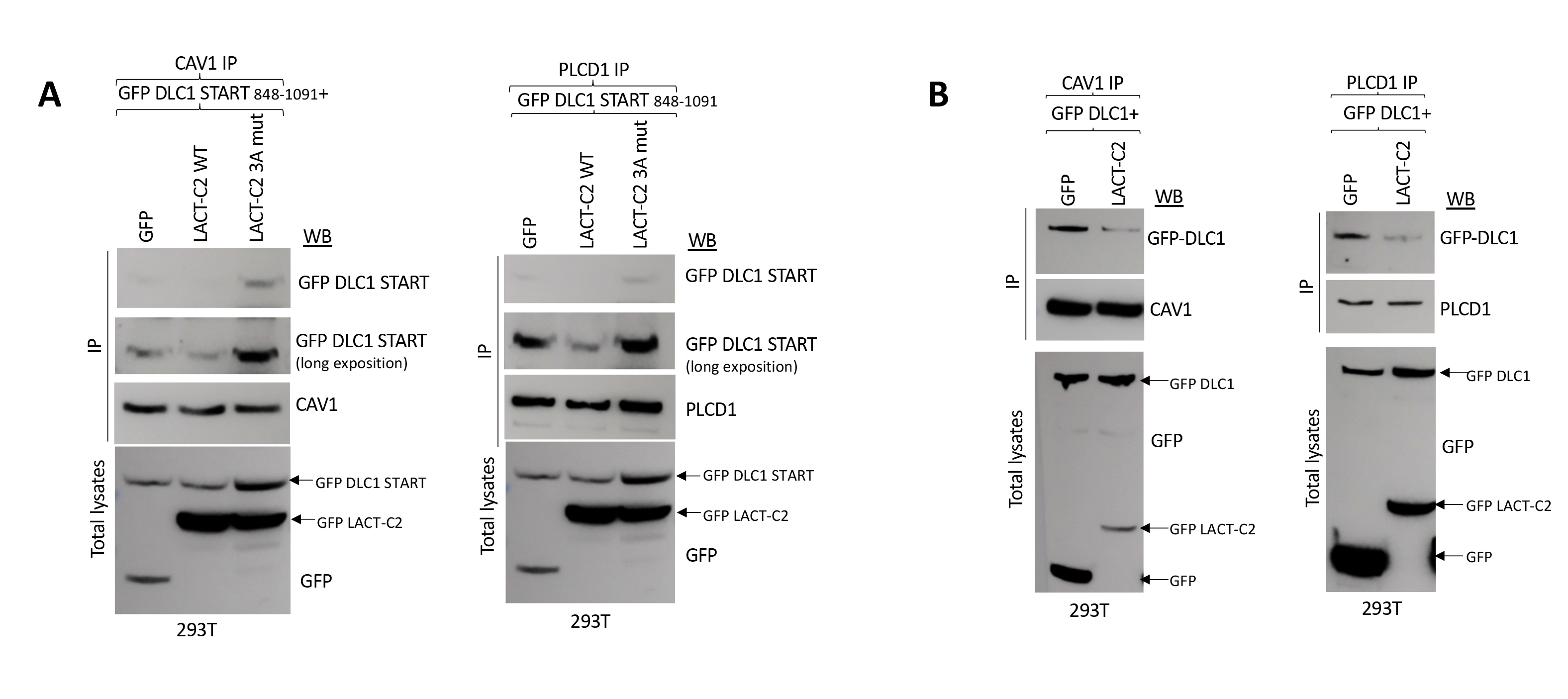

Supplement: Supplementary file 1 — Additional file 1. [file 12943_2021_1439_MOESM1_ESM.zip › Suppl Fig 4.png]

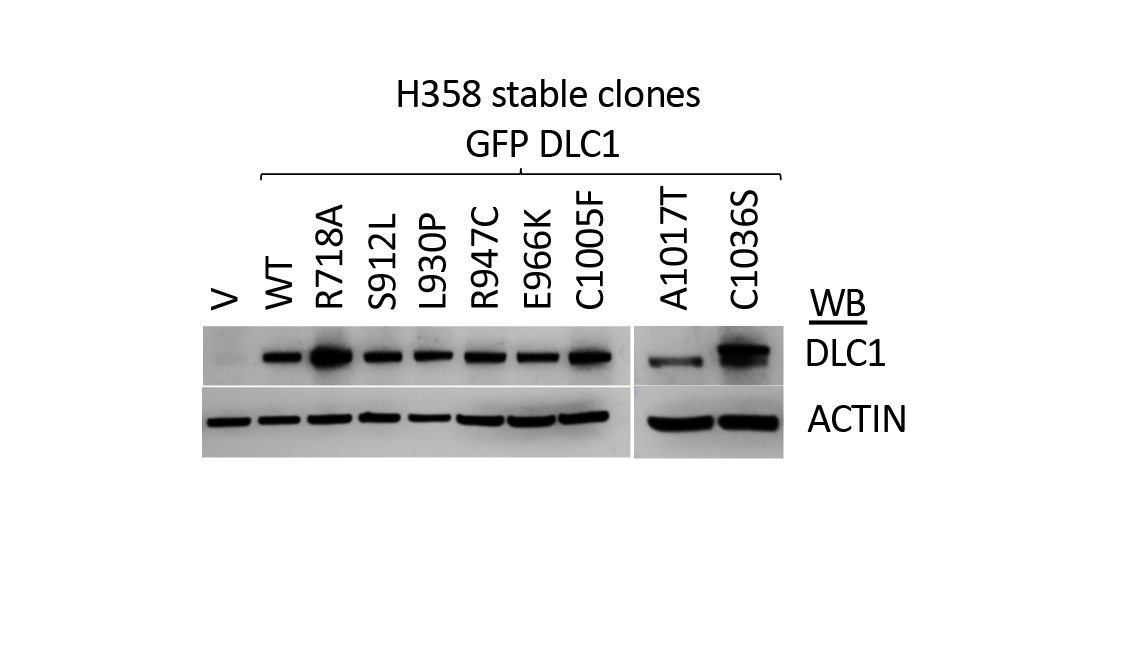

Supplement: Supplementary file 1 — Additional file 1. [file 12943_2021_1439_MOESM1_ESM.zip › Suppl fig 5.png]

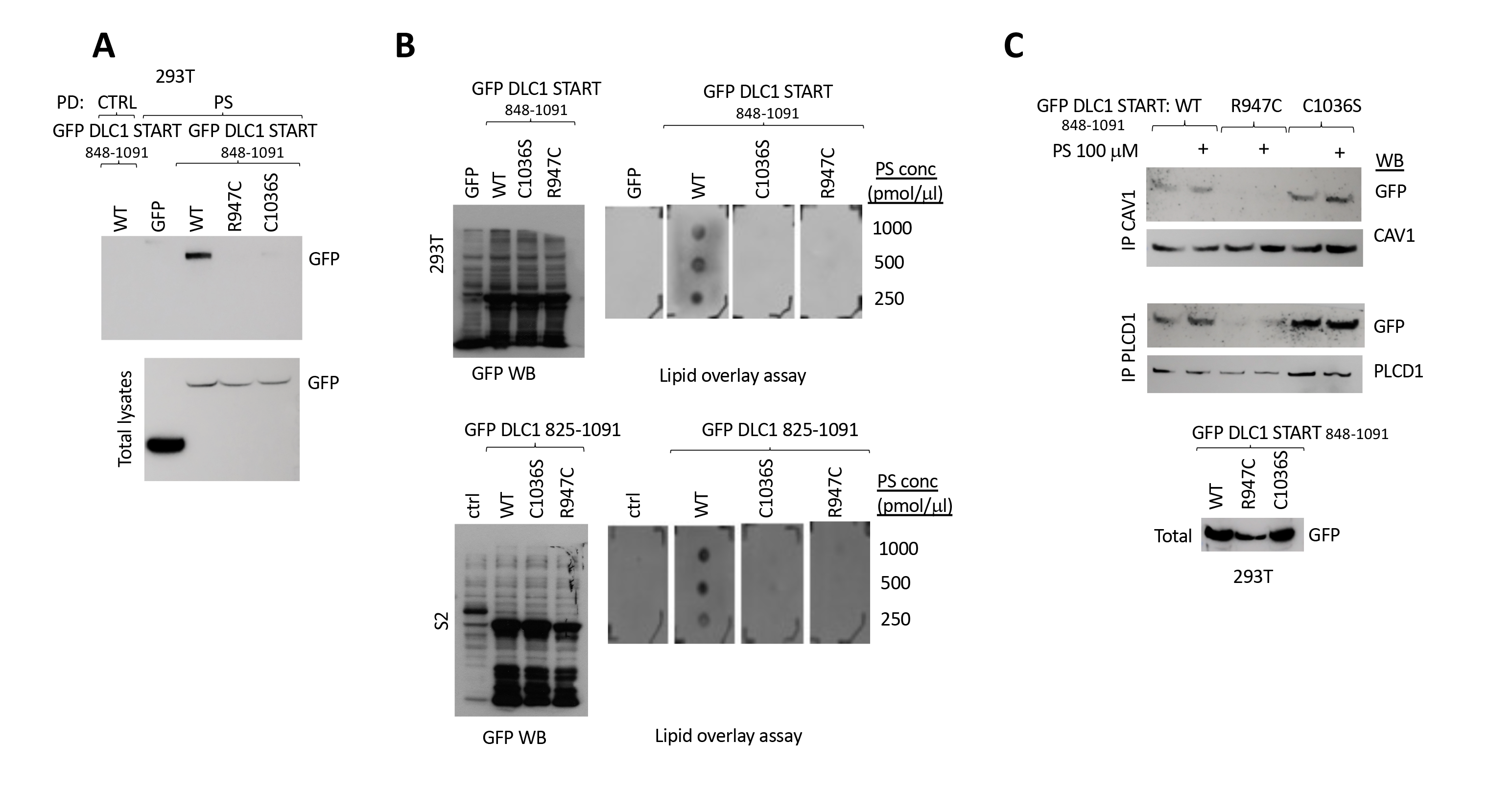

Supplement: Supplementary file 1 — Additional file 1. [file 12943_2021_1439_MOESM1_ESM.zip › Suppl fig 6.png]

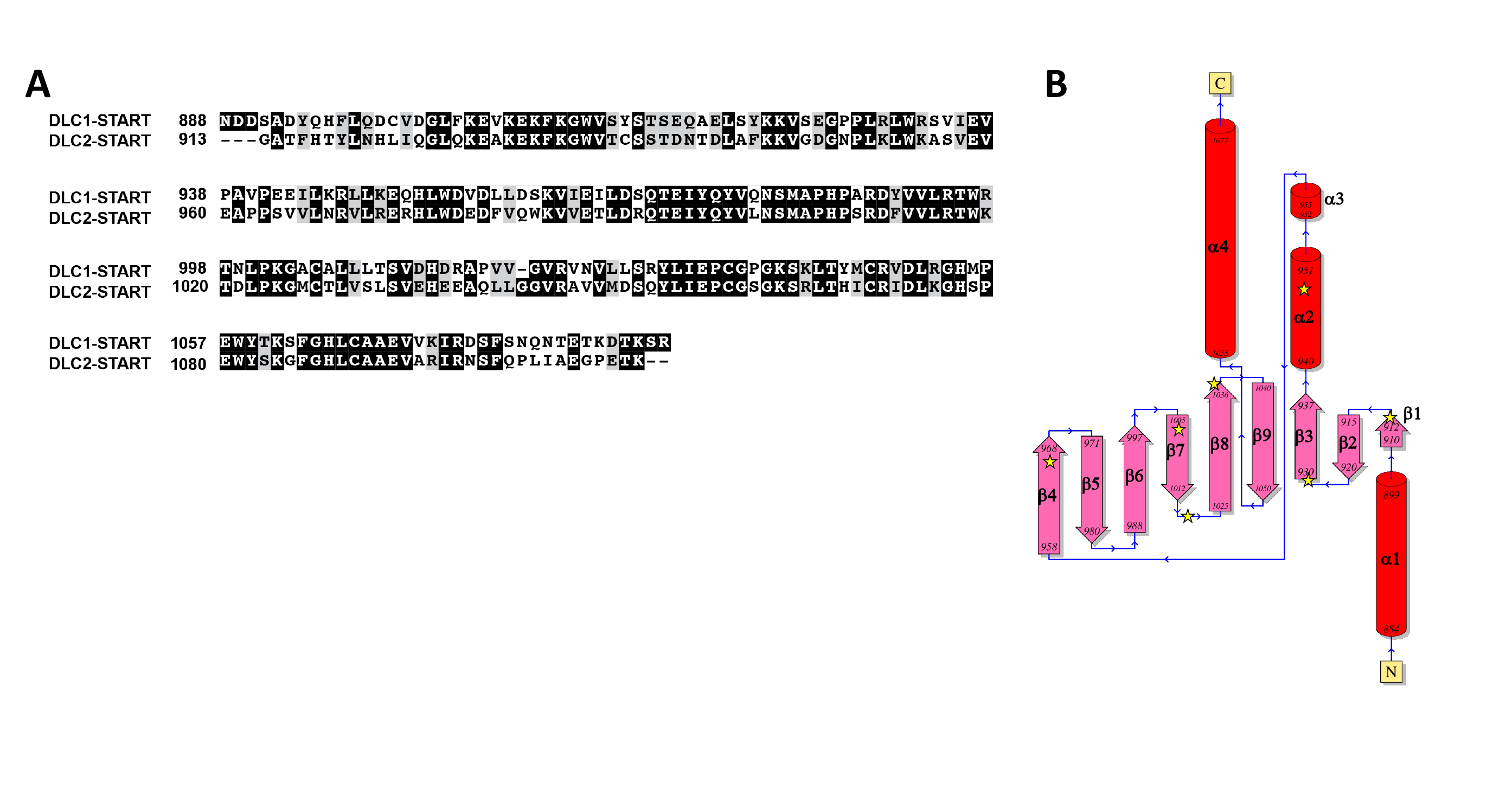

Supplement: Supplementary file 1 — Additional file 1. [file 12943_2021_1439_MOESM1_ESM.zip › Suppl fig 7.png]

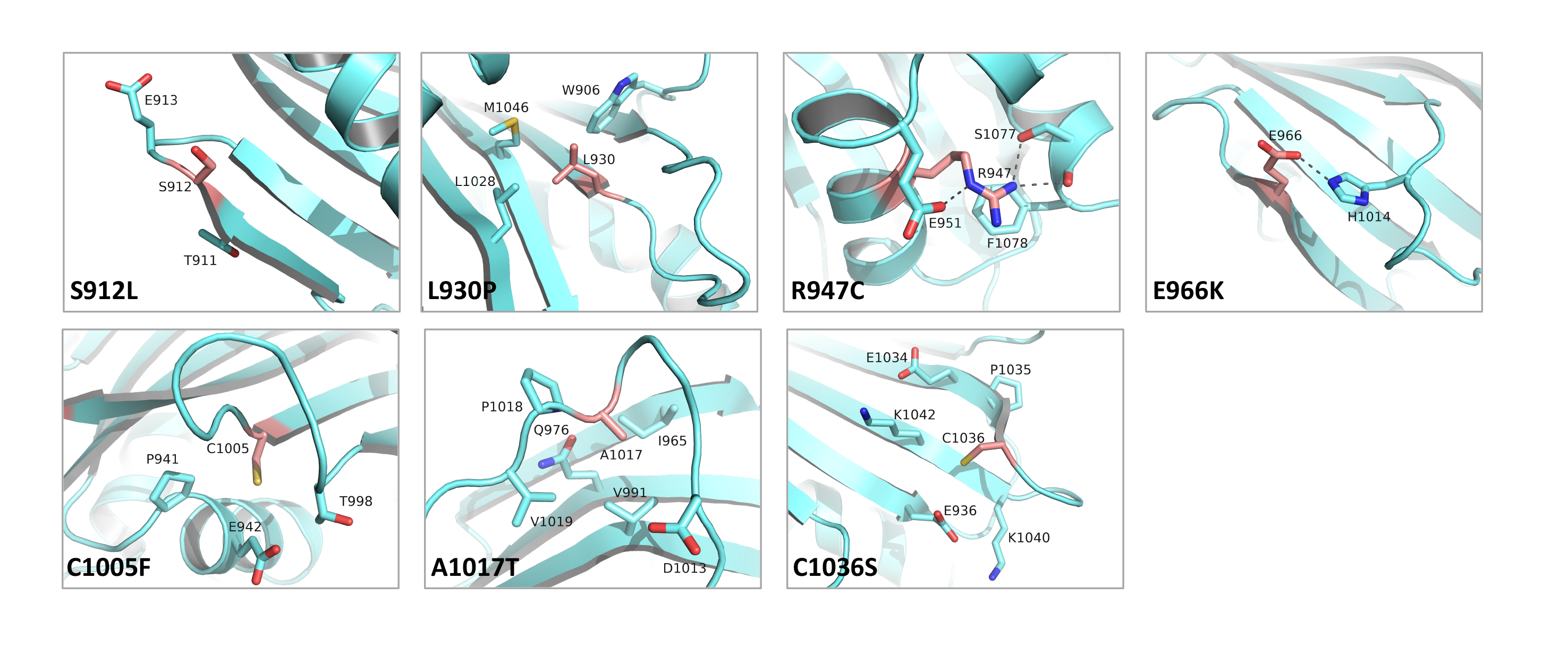

Supplement: Supplementary file 1 — Additional file 1. [file 12943_2021_1439_MOESM1_ESM.zip › Suppl fig 8.png]
